# New methods for separating causes from effects in genomics data

**DOI:** 10.1186/1471-2164-13-S8-S22

**Published:** 2012-12-17

**Authors:** Alexander Statnikov, Mikael Henaff, Nikita I Lytkin, Constantin F Aliferis

**Affiliations:** 1 Center for Health Informatics and Bioinformatics, New York University Langone Medical Center, New York, NY 10016, USA; 2 Department of Medicine, Division of Translational Medicine, New York University School of Medicine, New York, NY 10016, USA; 3 Department of Pathology, New York University School of Medicine, New York, NY 10016, USA; 4 Department of Biostatistics, Vanderbilt University, Nashville, TN, 37232, USA

## Abstract

**Background:**

The discovery of molecular pathways is a challenging problem and its solution relies on the identification of causal molecular interactions in genomics data. Causal molecular interactions can be discovered using randomized experiments; however such experiments are often costly, infeasible, or unethical. Fortunately, algorithms that infer causal interactions from observational data have been in development for decades, predominantly in the quantitative sciences, and many of them have recently been applied to genomics data. While these algorithms can infer unoriented causal interactions between involved molecular variables (i.e., without specifying which one is the cause and which one is the effect), causally orienting all inferred molecular interactions was assumed to be an unsolvable problem until recently. In this work, we use transcription factor-target gene regulatory interactions in three different organisms to evaluate a new family of methods that, given observational data for just two causally related variables, can determine which one is the cause and which one is the effect.

**Results:**

We have found that a particular family of causal orientation methods (IGCI Gaussian) is often able to accurately infer directionality of causal interactions, and that these methods usually outperform other causal orientation techniques. We also introduced a novel ensemble technique for causal orientation that combines decisions of individual causal orientation methods. The ensemble method was found to be more accurate than any best individual causal orientation method in the tested data.

**Conclusions:**

This work represents a first step towards establishing context for practical use of causal orientation methods in the genomics domain. We have found that some causal orientation methodologies yield accurate predictions of causal orientation in genomics data, and we have improved on this capability with a novel ensemble method. Our results suggest that these methods have the potential to facilitate reconstruction of molecular pathways by minimizing the number of required randomized experiments to find causal directionality and by avoiding experiments that are infeasible and/or unethical.

## Background

The discovery of molecular pathways that drive diseases and vital cellular functions is a fundamental activity of biomedical research. Unraveling disease pathways is a major component in the efforts to develop new therapies that will effectively fight deadly diseases. Furthermore, knowing pathways significantly facilitates the design of personalized medicine modalities for diagnosis, prognosis, and management of diseases. The discovery of pathways is a challenging problem and its solution to a large extent relies on the identification of *causal *molecular interactions in genomics data.

By causal molecular interactions or relations we mean interactions of molecular variables that match the notion of randomized controlled experiment, which is the de facto standard for assessing causation in the general sciences and biomedicine [1-5]. Assume that a hypothetical experimenter can change the distribution of a variable X (i.e., experimentally manipulate it). We say that X is a cause of Y (and Y is an effect of X) and denote this by X→Y if the probability distribution of Y changes for some experimental manipulation of X.

Causal molecular interactions can be discovered using randomized experiments such as interference with RNA (e.g., shRNA, siRNA); however such experiments are often costly, infeasible, or unethical. Fortunately, over the last 20 years many algorithms that infer causal interactions from *observational *data have been developed [1-5] and some of them have been adopted to the high dimensionalities of modern genomics data [6,7]. Outside of biomedicine, two Nobel prizes have recently been awarded in 2003 and 2011 for methods which seek to discover causal relations from non-experimental data [8-11].

In our prior work we evaluated the ability of state-of-the-art causal discovery algorithms to de-novo identify *unoriented *edges in genome-scale regulatory networks [12], which represent causal interactions between transcription factors and their target genes without distinguishing the mechanistic role of the involved molecular variables (i.e., we did not assess which genes were transcription factors and which genes were their targets). We deliberately avoided performing causal orientation of the discovered unoriented edges (i.e., separating transcription factors/causes from their target genes/effects) because this problem has previously been deemed worst-case unsolvable in observational data using existing algorithms [1] due to the statistical indistinguishability of causal models in the same Markov equivalence class. For example, causal models X→Y and X←Y have generally been assumed to be statistically indistinguishable given only observational data for X and Y.

Over the last 5 years researchers have developed a new class of methods that, given observational data for just two causally related variables X and Y, aim to determine which variable is the cause and which one is the effect (e.g., separate X→Y from X←Y) [13-18]. These causal orientation methods aim to solve the problem of statistical indistinguishability of graphs in the Markov equivalence class by *exploiting asymmetries in the shapes of the conditional probability densities *and *without requiring randomized experiments*. These methods could have significant implications for the field of causal discovery because they can orient unoriented edges that are discoverable by other established techniques, e.g. Generalized Local Learning (GLL) or Local-to-Global Learning (LGL) [6,7]. Therefore, at face value, these causal orientation methods have the potential to reduce the number of, and in some cases even eliminate, randomized experiments needed for causal orientation of edges in the Markov equivalence class of graphs and make the causal model fully identifiable from observational data alone.

As promising as these new causal orientation methods are, they have not been previously applied in genomics, where the data is usually noisy and the sample sizes are relatively small compared to prior test applications of these methods [13-18]. In this paper we report results of an extensive study of recent causal orientation techniques in the genomics domain by (i) testing 12 methods/variants to distinguish cause (in our experiments, transcription factor) from effect (in our experiments, target gene) in 5,739 causal interactions and (ii) conducting sensitivity analyses with respect to noise and sample size for the best-performing methods. In addition, (iii) we introduce a new ensemble technique for causal orientation that is shown to be more accurate than any best individual causal orientation method in the tested data. The results of this study can serve as a foundation for further development of causal orientation techniques for genomics data and establishing a context for wide applications in molecular or biomedical research.

## Methods

### Causal orientation methods

As mentioned above, the purpose of the tested causal orientation methods is to separate cause from effect given data for just two variables X and Y *that have a causal relation *(i.e., in the underlying data generative distribution, either X → Y or X ← Y). Typically these methods are not designed to be used to causally orient pairs of variables that only have univariate association/correlation due to the possibility of confounding. For example, in the majority of distributions, a causal structure X ← T → Y implies that X and Y are associated even though they are not causally affecting each other. Therefore, the presence of association in a pair of variables X and Y is, in general, a necessary but not sufficient condition to be eligible for causal orientation. A rigorous approach would involve first using correct causal discovery methods (e.g., GLL/LGL [6,7], MMHC [19], PC/FCI [1], IC/IC* [2], etc.) to identify unoriented edges that denote the existence of unconfounded causal relations and then applying causal orientation techniques to orient unoriented edges.

While each causal orientation method has its own principles and sufficient assumptions that are outlined in Table 1 (and brief descriptions of the algorithms are given in the Additional file 1), most of these techniques are based on the idea that the factorization of the joint probability distribution P(cause,effect) into P(cause)P(effect|cause) yields a simpler representation than the factorization into P(effect)P(cause|effect). One can furthermore show that if the marginal probability distribution of the cause P(cause) is independent of the causal mechanism P(effect|cause), then the factorization P(cause)P(effect|cause) has lower complexity than the factorization P(effect)P(cause|effect). Given two causally related variables X and Y, estimating the complexity of the two different factorizations of P(X,Y) or determining independence between marginal and conditional distributions can thus provide the basis for causal orientation techniques. In practice, however, it is difficult to directly test independence between P(X) and P(Y|X) or estimate (or even define a measure of) their complexity; hence the methods typically use simplifying assumptions or rely on approximate formulations.

**Table 1:** *High-level description of the tested causal orientation methods*.

Method	Reference	Key principles	Sufficient assumptions for causally orienting X → Y	Sound
**ANM**	[14]	Assuming X → Y with Y = f(X) + e_1_, where X and e_1 _are independent, there will be no such additive noise model in the opposite direction X ← Y, X = g(Y) + e_2_, with Y and e_2 _independent.	• Y = f(X) + e_1_; • X and e_1 _are independent; • f is non-linear, or one of X and e is non-Gaussian; • Probability densities are strictly positive; • All functions (including densities) are 3 times differentiable.	Yes
**PNL**	[15]	Assuming X → Y with Y = f_2_(f_1_(X) + e_1_), there will be no such model in the opposite direction X←Y, X = g_2_(g_1_(Y) + e_2_) with Y and e_2 _independent.	• Y = f_2_(f_1_(X) + e_1_); • X and e_1 _are independent; • Either f_1 _or e_1 _is Gaussian; • Both f_1 _and f_2 _are continuous and invertible.	Yes
**IGCI**	[16,17]	Assuming X→Y with Y = f(X), one can show that the KL-divergence (a measure of the difference between two probability distributions) between P(Y) and a reference distribution (e.g., Gaussian or uniform) is greater than the KL-divergence between P(X) and the same reference distribution.	• Y = f(X) (i.e., there is no noise in the model); • f is continuous and invertible; • Logarithm of the derivative of f and P(X) are not correlated.	Yes
**GPI-MML**	[18]	Assuming X→Y, the least complex description of P(X, Y) is given by separate descriptions of P(X) and P(Y|X). By estimating the latter two quantities using methods that favor functions and distributions of low complexity, the likelihood of the observed data given X→Y is inversely related to the complexity of P(X) and P(Y | X).	• Y = f(X, e); • X and e are independent; • e is Gaussian; • The prior on f and P(X) factorizes.	No
**ANM-MML**	[18]	Same as for GPI-MML, except for a different way of estimating P(Y | X) and P(X | Y).	• Y = f(X) + e; • X and e are independent; • e is Gaussian. • The prior on f and P(X) factorizes.	No
**GPI**	[18]	Assuming X→Y with Y = f(X,e_1_), where X and e_1 _are independent and f is "sufficiently simple", there will be no such model in the opposite direction X←Y, X = g(Y,e_2_) with Y and e_2 _independent and g "sufficiently simple".	Same as for GPI-MML.	No
**ANM-GAUSS**	[18]	Same as for ANM-MML, except for the different way of estimating P(X) and P(Y).	Same as for ANM-MML.	No
**LINGAM**	[13]	Assuming X→Y, if we fit linear models Y = b_2_X+e_1 _and X = b_1_Y+e_2 _with e_1 _and e_2 _independent, then we will have b_1 _< b_2_.	• Y = b_2_X+e_1_; • X and e_1 _are independent; • e_1 _is non-Gaussian.	Yes

The last column indicates whether a method is sound, i.e. it can provably orient a causal structure under its sufficient assumptions. Because causal orientation methodologies are fairly new and not completely characterized, it is possible that proofs of correctness will become available for GPI-MML, ANM-MML, GPI, and ANM-GAUSS. All methods implicitly assume that there are no feedback loops. The noise term in the models is denoted by small "e".

The majority of tested causal orientation methods (IGCI, LINGAM, GPI-MML, ANM-MML, ANM-GAUSS) output two scores indicating likelihood of the forward causal model (X → Y) and the backward one (X ← Y). Other tested methods (ANM, PNL, GPI) output two p-values indicating significance of the forward and backward causal models. In order to make all methods directly comparable to each other, we decided to force them to make causal orientation decisions for all tested causal interactions. This was achieved by comparing scores or p-values of the forward and backward causal models and selecting the most likely orientation. This approach follows the practices of previously published applications of causal orientation methods by their inventors [16-18].

We also note that an alternative approach for the ANM, PNL, and GPI methods is to select a model (forward/backward) that is statistically significant at a given alpha level. The latter approach can sometimes improve accuracy of the causal orientation method while reducing the fraction of causally oriented edges [17]. While results for this approach are not central to this manuscript, we report them in the Additional file 1. We also note that the main findings for the primary approach are for the most part consistent with the alternative approach.

Finally, prior to application of the causal orientation methods, we standardized the data to mean zero and standard deviation one.

### Gold standard construction and observational data

The primary challenges in evaluating causal orientation methods for genomics applications are (i) limited availability of known gold standards of causal molecular interactions and (ii) limited sample sizes of the available observational data. To overcome these challenges we focused on transcription factor-gene regulatory interactions that have been discovered on the genome wide level and experimentally verified in model organisms [20,21] and more recently in human cell lines [22]. Therefore, the gold standards in this work contain tuples of genes (X, Y) with orientation X→Y, where X is a transcription factor and Y is its target gene.

We used the following four gold standards: (i) interactions of the NOTCH1 transcription factor and its target genes in human T-cell acute lymphoblastic leukaemia (denoted as NOTCH1); (ii) interactions of the RELA transcription factor and its target genes in human T-cell acute lymphoblastic leukaemia (denoted as RELA); (iii) interactions of 140 transcription factors and their target genes in Escherichia coli (denoted as ECOLI); and (iv) interactions of 115 transcription factors and their target genes in Saccharomyces cerevisiae (denoted as YEAST). We used microarray gene expression data from the public domain for orientation of causal relations in each gold standard. The summary statistics of gold standards and corresponding microarray gene expression datasets are given in Table 2 and Table 3, and details of gold standard creation are provided below.

**Table 2:** *Information about gold standards (GS) used in the study*.

Task name	Reference/source	# TFs in GS	# genes in GS	# gene probes for GS genes in gene expression data	# TF-gene interactions	# TF-gene interactions significant at FDR = 0.05
***ECOLI***	[12,20]	140	913	913	1,885	***1,607***
***YEAST***	[12,21]	115	1,834	1,834	3,541	***2,648***
***NOTCH1***	[22,40]	1	302	813	813	***553***
***RELA***	[22,41,42]	1	1,420	3,657	3,657	***931***

"TF" stands for "transcription factor". Statistically significant associations were determined using Fisher's Z-test at 5% FDR in microarray gene expression data (please see text for details).

**Table 3:** *Information about microarray gene expression datasets used in the study for each gold standard*.

Task name	Reference/source	# samples
***ECOLI***	[43]	907
***YEAST***	[43]	530
***NOTCH1***	[44]	174
***RELA***	[44]	174

Only T-ALL samples were selected for NOTCH1 and RELA in order to match cell population used for creation of the respective gold standard.

Once each of the gold standards was constructed, we removed interactions without statistically significant associations (in the observational data) according to Fisher's Z-test [23] at 5% FDR [24,25]. We performed this filtering because presence of association is a necessary condition for detecting causal relations in most practical settings.

All gold standards and microarray gene expression datasets are available for download from http://www.nyuinformatics.org/downloads/supplements/CausalOrientation.

***Creation of NOTCH1 and RELA gold standards***: These gold standards contain genes that are directly downstream of a particular transcription factor (NOTCH1 or RELA) and are functionally regulated by it. The gold standards were obtained using the method described in [22].

Functional gene expression data was first used to identify genes that are downstream (but not necessarily directly) of a particular transcription factor. The samples in such data are randomized to either 'experiment' (e. g., siRNA knockdown of the transcription factor of interest) or 'control' treatment. All genes that are differentially expressed between 'experiment' and 'control' treatments are expected to be downstream of the transcription factor. We have used a t-test with α = 0.05 to identify such genes.

Genome-wide binding data (ChIP-on-chip for NOTCH1 and ChIP-sequencing for RELA) was then employed to identify direct binding targets of each transcription factor. Specifically, for each studied transcription factor we obtained the set of genes with detected promoter region-transcription factor binding according to the primary study that generated binding data.

We note that using genome-wide binding data by itself is insufficient to find downstream functional targets of a transcription factor, because many binding sites can be non-functional [26]. Therefore, the final step in gold standard creation required overlapping the list of direct binding targets (from binding data) with the list of downstream functional targets (from gene expression data). Knowledge gained by integration of data from these two sources is believed to provide high confidence that a given transcription factor directly regulates a particular gene [27]. Also, integration of data from two different sources contributes to the reduction of false positives in the resulting gold standards.

***Creation of gold standard for YEAST and ECOLI: ***These gold standards contain genes that bind to and are likely to be regulated by the known transcription factors in Saccharomyces cerevisiae and Escherichia coli.

The Saccharomyces cerevisiae (denoted as YEAST) gold standard was built by identifying the promoter sequences that are both bound by transcription factors according to ChIP-on-chip data at 0.001 alpha level and conserved within 2 related species in the Saccharomyces genus [12,21]. Binding information is essential because transcription factors must first bind to a gene to induce or suppress expression, while conservation information is important because true-positive transcription factor-DNA interactions are often conserved within a genus.

The Escherichia coli (denoted as ECOLI) gold standard was constructed from RegulonDB (version 6.4), a manually curated database of regulatory interactions obtained mainly through a literature search [12,20]. ChIP-qPCR data has shown RegulonDB to be approximately 85% complete [28,29]. Evidence for each regulatory interaction in RegulonDB is classified as "strong" or "weak", depending on the type of experiment used to predict the interaction. For example, binding of a transcription factor to a promoter is considered strong evidence, whereas gene-expression based computational predictions are considered weak evidence. For the purposes of our study, we created a conservative gold standard of only strong interactions.

The gold standards YEAST and ECOLI contain relations of the type "transcription factor → gene" and "transcription factor → transcription factor" (where "gene" refers to a target gene that is not a transcription factor). We decided to simplify the setting of our evaluation when we assess whether the inferred causal orientation X→Y or X←Y is correct, and restricted attention to only interactions of the type "transcription factor → gene". This results in minimizing the number of cases with feedback that can be represented by causal edges in both directions. Note that it is not currently possible to comprehensively apply this filtering step to NOTCH1 and RELA gold standards because the transcription factors are not well known in human cells.

### Performance metrics and statistical significance testing

Two metrics were used to assess performance of causal orientation algorithms. The first metric is accuracy which is the percentage of causal interactions that have been oriented correctly. A method that orients all causal interactions in the gold standard as "transcription factor → gene" would achieve an accuracy of 1; a method that orients all interactions as "gene → transcription factor" would achieve an accuracy of 0; and a method that flips a fair coin to make every orientation decision would on average achieve an accuracy of 0.5.

The second metric is area under ROC curve (AUC), which is known to be more discriminative than accuracy because it takes into account the confidence of orientation decisions [30,31]. The ROC curve is the plot of sensitivity versus 1-specificity for a range of threshold values on the difference between the scores/p-values of the forward and backward causal models [32]. AUC ranges from 0 to 1, where AUC = 1 corresponds to perfectly correct prediction of causal orientation, AUC = 0.5 corresponds to prediction by chance, and AUC = 0 corresponds to completely incorrect prediction of causal orientation. Computation of sensitivity/specificity and AUC requires a response variable with both positive and negative labels. We created such a response variable by representing gold standard edges (that always point from a transcription factor to its target gene) in the following two equivalent ways: 50% of the edges were represented as "transcription factor → gene" and the other 50% were represented as "gene ← transcription factor". The edges "→" were labeled as positives and "←" were labeled as negatives. This process is illustrated in Table 4; in particular note that the direction of causality always points from transcription factor to gene. AUC was then computed according to the formula given in [33], with the difference in scores/p-values serving as a predictor. Note that the AUC can also be interpreted as the probability that the difference between scores/p-values of the forward and backward causal models for a randomly chosen positive instance is higher than the difference between scores/p-values for a randomly chosen negative instance. Since each of the four gold standards has a large number of edges (>500), the variance in AUC due to different choices of edges for negative and positive labels is minimal and typically smaller than 0.001 AUC, as estimated by computation of AUC for 1,000 random choices of positive and negative labels.

**Table 4:** An example demonstrating the construction of the response variable for AUC computation

a)			b)			
**Variable 1**	**Causal Edge**	**Variable 2**	**Variable 1**	**Causal Edge**	**Variable 2**	**Response**

NOTCH1	→	ABCF2	NOTCH1	→	ABCF2	**+**
NOTCH1	→	EIF4E	**EIF4E**	**←**	**NOTCH1**	**-**
NOTCH1	→	SFRS3	NOTCH1	→	SFRS3	**+**
NOTCH1	→	NUP98	**NUP98**	**←**	**NOTCH1**	**-**
NOTCH1	→	CYCS	NOTCH1	→	CYCS	**+**
NOTCH1	→	ZNHIT	**ZNHIT**	**←**	**NOTCH1**	**-**
NOTCH1	→	ATM	NOTCH1	→	ATM	**+**
NOTCH1	→	TIMM9	**TIMM9**	**←**	**NOTCH1**	**-**

A fragment of the gold standard is shown in a). The edges always point from a transcription factor (NOTCH1) to its target gene. 50% of the edges are represented as "transcription factor → gene" and the other 50% as "gene ← transcription factor" in b). This constructs a response variable with positives corresponding to "→" edges (shown in black) and negatives corresponding to "←" edges (shown in red).

**Figure 1:**
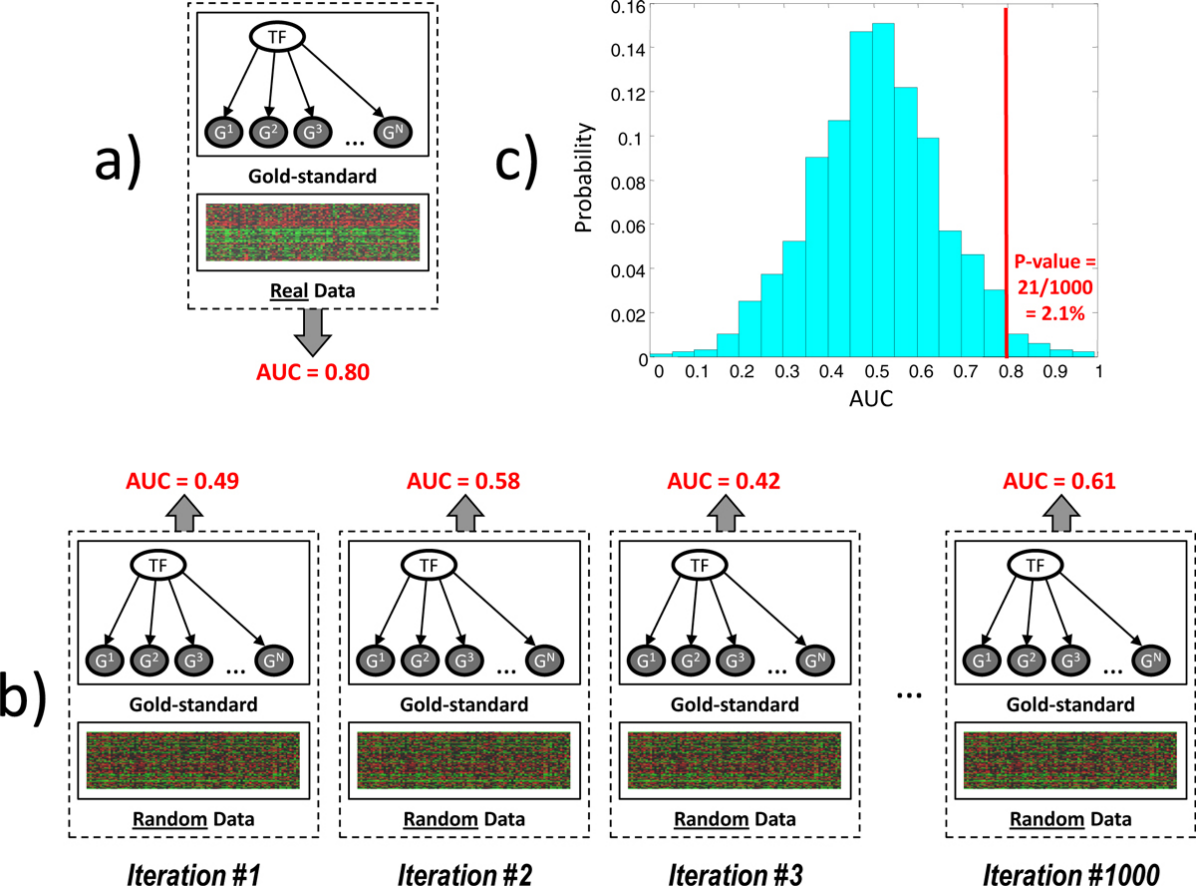
***Illustration of the statistical testing procedure to assess significance of the causal orientation method performance (AUC/accuracy)***. a) AUC is computed using real data; b) AUC is computed using random data from the Normal distribution (null distribution) for the same gold standard as used with the real data, and this step is repeated 1,000 times; c) a p-value is calculated by comparing AUCs from the null distribution to the AUC obtained in the real data.

Given values of the above two performance metrics (accuracy and AUC), we need to assess their statistical significance, i.e. find out if the causal orientation is better than by chance. Notice that our gold standards are such that many causal edges are not independent because they share the same transcription factor. That is why we chose to apply an exact statistical testing procedure that can accurately estimate the variance of orientation by chance in our setting [34]. A schematic illustration of the statistical testing procedure is given in Figure 1. First we compute AUC using real gene expression data (Figure 1-a). Then we replace the real data with random data from the Normal distribution with mean 0 and standard deviation 1 (the null distribution) and compute AUC for the *same gold standard *as used with the real data. This step is repeated with 1,000 different randomly generated datasets (Figure 1-b). Finally, we compare AUCs from the null distribution to the AUC obtained in the real data and compute a p-value that corresponds to the proportion of random datasets that yield higher AUCs than the real data (Figure 1-c). A downside of the above statistical testing procedure is that it is computationally expensive and requires running each causal orientation method 5,744,739 times (= 5,739 causal interaction · 1,001 datasets) in order to assess its significance in all 4 gold standards used in our study. To make this analysis feasible, we assessed the statistical significance of only the two best performing methods (IGCI Gaussian/Entropy and IGCI Gaussian/Integral) and utilized the Asclepius Compute Cluster at the Center for Health Informatics and Bioinformatics (CHIBI) at New York University Langone Medical Center (http://www.nyuinformatics.org).

### Methodology for sensitivity analyses

In order to study sensitivity to sample size (number of observations), we sample without replacement from the original gene expression data nested subsets of size 10, 20, 30, ..., N, where N is the number of samples in the dataset. Specifically, the subset of size 10 is included in the subset of size 20, which is in turn included in the subset of size 30, and so on. We then run the causal orientation algorithms on each subset and compute performance. This process is repeated with different sampled nested subsets, and mean performance and variance are estimated over all runs. For the NOTCH1 and RELA gold standards, we used 100 subsets of each size, while for the more computationally expensive YEAST and ECOLI gold standards we used 20 subsets of each size.

For the sensitivity analysis to noise, we add a certain proportion (p) of random Gaussian noise to the gene expression data for both transcription factors and their target genes, run causal orientation methods in the noisy data, report their performance, and repeat the entire process to assess variance (again, 100 times for NOTCH1 and RELA and 20 times for YEAST and ECOLI). Denoting by X the transcription factor and by Y its target gene, the noisy transcription factor X′ and gene Y′ are defined as follows: X′ = (1-p) · X + p · N(M_X_,S_X_) and Y′ = (1-p) · Y + p · N(M_Y_,S_Y_), where N(m,s) is a Normally distributed random variable with mean m and standard deviation s, and M_X_, M_Y _and S_X_, S_Y _are means and standard deviations of X and Y in the original data (prior to noise addition). We use the following proportions of noise (values of p): 0.05, 0.10, 0.15, ..., 0.90, 0.95, 1.00.

### A new ensemble method for causal orientation

As an enhancement to using individual causal orientation techniques, we introduce ensemble causal orientation models that combine decisions of all available individual causal orientation methods in order to produce a more powerful predictor of causal orientation. These methodologies are popular in the field of supervised learning, where non-random weak learners are often combined to produce a more accurate predictor [35]. The use of ensemble modelling for causal orientation is motivated by our empirical observations that there is no single causal orientation methodology that performs perfectly (i.e., with 1.0 accuracy or AUC), many causal orientation methods appear to perform different than chance, and causal orientation methods often make errors in orienting *different *edges.

In this study we experimented with a straightforward approach to ensemble modelling, where we train a logistic regression model [36] on predictions of all 11 tested causal orientation methods (i.e., the input features consist of the differences in scores/p-values between forward and backward models). For the response variable, we follow the same approach as for the computation of AUC which was described above in the subsection on performance metrics. Namely, 50% of edges are represented as "transcription factor → gene" and the other 50% as "gene ← transcription factor". Then the response variable is constructed by labelling "→" edges as positives and "←" edges as negatives.

As with every supervised learning procedure that is trained and tested using the same dataset, it is essential to split the available data into non-overlapping training and testing sets, whereby the training set is used to fit a learning model and the testing set is used to estimate its performance [37,38]. For each gold standard we used 30% of the causal interactions (chosen at random) for training and the remaining 70% for testing. We decided to use a training set that was smaller than the testing set so that our study resembles a possible practical application where only a small portion of the gold standard is known and the rest is to be discovered. The predictions of the ensemble model in each gold standard are compared with the predictions of the best individual causal orientation technique in the same testing set with 70% of the data (to ensure that the results are directly comparable).

Finally, in addition to exploring holdout validation performance of the ensemble models, we trained and tested the ensemble models on different gold standards. In practice this approach can be justified if the data distributions in the gold standards used for training and testing of ensemble models are similar. It also resembles a practical situation when a gold standard is known in a previously studied dataset but is not known in a new but distributionally similar one.

## Results

### Evaluating causal orientation methods with the accuracy metric

The causal orientation accuracy values are given in Table 5 for 12 causal orientation methods (including orientation by flipping a fair coin which is denoted as "RANDOM" in the table) and 4 gold standards. The performance ranks of methods with accuracies higher than 0.50 are given in Table 6.

**Table 5:** Accuracy of causal orientation

Method	ECOLI	YEAST	NOTCH1	RELA
ANM	0.462	0.383	0.476	0.396
PNL	0.453	0.471	**0.521**	**0.520**
IGCI (Uniform/Entropy)	**0.647**	0.427	**0.611**	**0.692**
IGCI (Uniform/Integral)	**0.605**	0.441	**0.561**	**0.669**
IGCI (Gaussian/Entropy)	**0.742**	**0.555**	**0.848**	**0.898**
IGCI (Gaussian/Integral)	**0.645**	**0.587**	**0.729**	**0.835**
GPI-MML	0.485	0.390	0.251	0.395
ANM-MML	0.428	0.316	0.183	0.172
GPI	**0.526**	0.401	**0.548**	**0.506**
ANM-GAUSS	0.480	0.483	**0.727**	0.462
LINGAM	0.469	0.451	0.367	0.387
RANDOM	0.500	0.500	0.500	0.500

For each gold standard (column) dark orange cells correspond to methods that have high values of accuracy, while white cells correspond to methods that have low values of accuracy. Accuracies higher than 0.50 are shown in bold.

**Table 6:** Ranks of causal orientation methods for each gold standard

Method	ECOLI	YEAST	NOTCH1	RELA
ANM	-	-	-	-
PNL	-	-	**5**	**5**
IGCI (Uniform/Entropy)	**2**	-	**3**	**3**
IGCI (Uniform/Integral)	**3**	-	**4**	**4**
IGCI (Gaussian/Entropy)	**1**	**2**	**1**	**1**
IGCI (Gaussian/Integral)	**2**	**1**	**2**	**2**
GPI-MML	-	-	-	-
ANM-MML	-	-	-	-
GPI	**4**	-	**4**	**5**
ANM-GAUSS	-	-	**2**	-
LINGAM	-	-	-	-

Ranks were computed only for the methods with accuracies greater than 0.50. The lower the rank, the better the accuracy of the causal orientation method for the given gold standard. The computation of rank took into account statistical variability, e.g. accuracies 0.647 and 0.645 obtained by the two IGCI methods in the ECOLI gold standard are statistically indistinguishable; that is why they have the same rank value.

As can be seen, IGCI Gaussian/Entropy and IGCI Gaussian/Integral methods achieve the highest accuracies in each of the four gold standards. In general, the other causal orientation methods perform worse, and some methods (e.g., ANM-MML) consistently prefer wrong decisions and have accuracies lower than 0.5.

Interestingly, if we consider the best performing method (IGCI Gaussian/Entropy) with the average rank 1.25, its results are statistically significant at alpha = 0.05 according to the exact test (described in the Methods section) only for the ECOLI gold standard (p-value < 0.001). The second best performing method (IGCI Gaussian/Integral) with the average rank 1.75 achieves significance in two out of four gold standards (p-value < 0.001 for ECOLI and 0.003 for YEAST) at alpha = 0.05. The reason why the IGCI Gaussian/Integral method achieves significance in more gold standards than the best performing technique IGCI Gaussian/Entropy is the small variance of the former method. The detailed statistical significance results including null distributions are given in the Additional file 1 in Figure S1 (for IGCI Gaussian/Entropy) and Figure S3 (for IGCI Gaussian/Integral). It is worth noting that statistical significance was achieved by neither of the two best performing methods in NOTCH1 and RELA gold standards that have only 1 transcription factor. This was primarily due to a large variance of causal orientation accuracies in the null distribution (see Figures S1 and S3 in the Additional file 1). On the other hand, if we join these two gold standards into one with 2 transcription factors, both methods achieve statistical significance at alpha = 0.05 (p-value of IGCI Gaussian/Entropy is 0.018 and p-value of IGCI Gaussian/Integral is 0.007).

The superior and often statistically significant performance of the two IGCI methods compared to other techniques was a surprising finding that we did not expect theoretically. IGCI assumes a noise free model (Table 1) that is unrealistic in genomics data. On the other hand, other methods that have *a priori *more realistic assumptions perform worse. We hypothesize that sufficient assumptions of the IGCI methods are too strict in practice and can be mitigated in many ways that are currently not well understood.

### Evaluating causal orientation methods with the AUC metric

The causal orientation AUC values are given in Table 7 for 12 causal orientation methods (including orientation by flipping a fair coin which is denoted as "RANDOM" in the table) and 4 gold standards. The performance ranks of methods with AUCs higher than 0.50 are given in Table 8.

**Table 7:** AUC of causal orientation

Method	ECOLI	YEAST	NOTCH1	RELA
ANM	0.464	0.379	0.456	0.369
PNL	0.443	0.464	**0.520**	**0.520**
IGCI (Uniform/Entropy)	**0.713**	0.409	**0.708**	**0.805**
IGCI (Uniform/Integral)	**0.642**	0.437	**0.631**	**0.757**
IGCI (Gaussian/Entropy)	**0.813**	**0.613**	**0.935**	**0.967**
IGCI (Gaussian/Integral)	**0.724**	**0.655**	**0.834**	**0.927**
GPI-MML	0.488	0.370	0.184	0.333
ANM-MML	0.393	0.237	0.078	0.071
GPI	**0.536**	0.396	**0.594**	**0.513**
ANM-GAUSS	0.474	0.476	**0.807**	0.446
LINGAM	0.462	0.463	0.362	0.392
RANDOM	0.500	0.500	0.500	0.500

For each gold standard (column) dark orange cells correspond to methods that have high values of AUC, while white cells correspond to methods that have low values of AUC. AUCs higher than 0.50 are shown in bold.

**Table 8:** Ranks of causal orientation methods for each gold standard

Method	ECOLI	YEAST	NOTCH1	RELA
ANM	-	-	-	-
PNL	-	-	**5**	**5**
IGCI (Uniform/Entropy)	**2**	-	**3**	**3**
IGCI (Uniform/Integral)	**3**	-	**4**	**4**
IGCI (Gaussian/Entropy)	**1**	**2**	**1**	**1**
IGCI (Gaussian/Integral)	**2**	**1**	**2**	**2**
GPI-MML	-	-	-	-
ANM-MML	-	-	-	-
GPI	**4**	-	**4**	**5**
ANM-GAUSS	-	-	**2**	-
LINGAM	-	-	-	-

Ranks were computed only for the methods with AUCs greater than 0.50. The lower the rank, the better the AUC of the causal orientation method for the given gold standard. The computation of rank took into account statistical variability, e.g. the AUCs of 0.724 and 0.713 obtained by the two IGCI methods in the ECOLI gold standard are statistically indistinguishable; that is why they have the same rank value.

Similarly to the accuracy results, IGCI Gaussian/Entropy and IGCI Gaussian/Integral methods achieve the highest AUCs in each of the four gold standards. Other causal orientation methods perform worse, and some methods (e.g., ANM-MML) consistently prefer wrong decisions and have AUCs lower than 0.5.

The statistical significance analysis of IGCI Gaussian/Entropy and IGCI Gaussian/Integral is described in detail in the Additional file 1 in Figure S2 (for IGCI Gaussian/Entropy) and Figure S4 (for IGCI Gaussian/Integral). In summary, both methods achieve statistical significance of causal orientations (at alpha = 0.05) in ECOLI and YEAST, only IGCI Gaussian/Integral achieves significance in RELA, and none of the two methods achieves significance in NOTCH1. On the other hand, similarly to results for the accuracy metric, both methods achieve statistically significant results in the joined NOTCH1 and RELA gold standard with 2 transcription factors.

### Sensitivity analysis to noise

**Figure 2:**
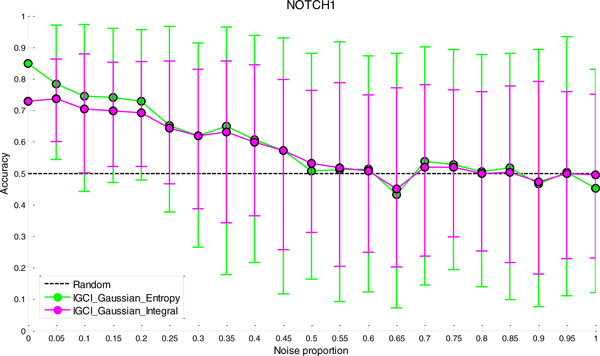
***Sensitivity analysis to noise in NOTCH1 gold standard for the best two IGCI methods***. Error bars denote 80% intervals of variation that were empirically estimated in 100 datasets for each value of the noise proportion.

**Figure 3:**
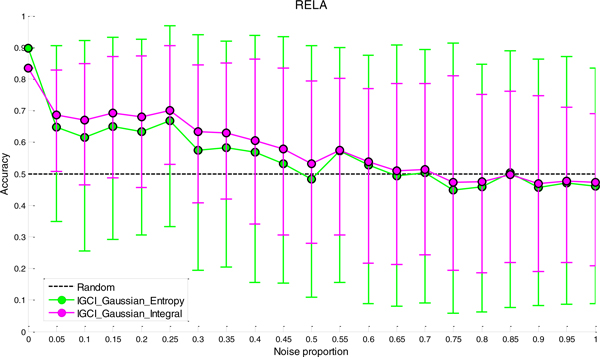
***Sensitivity analysis to noise in RELA gold standard for the best two IGCI methods***. Error bars denote 80% intervals of variation that were empirically estimated in 100 datasets for each value of the noise proportion.

**Figure 4:**
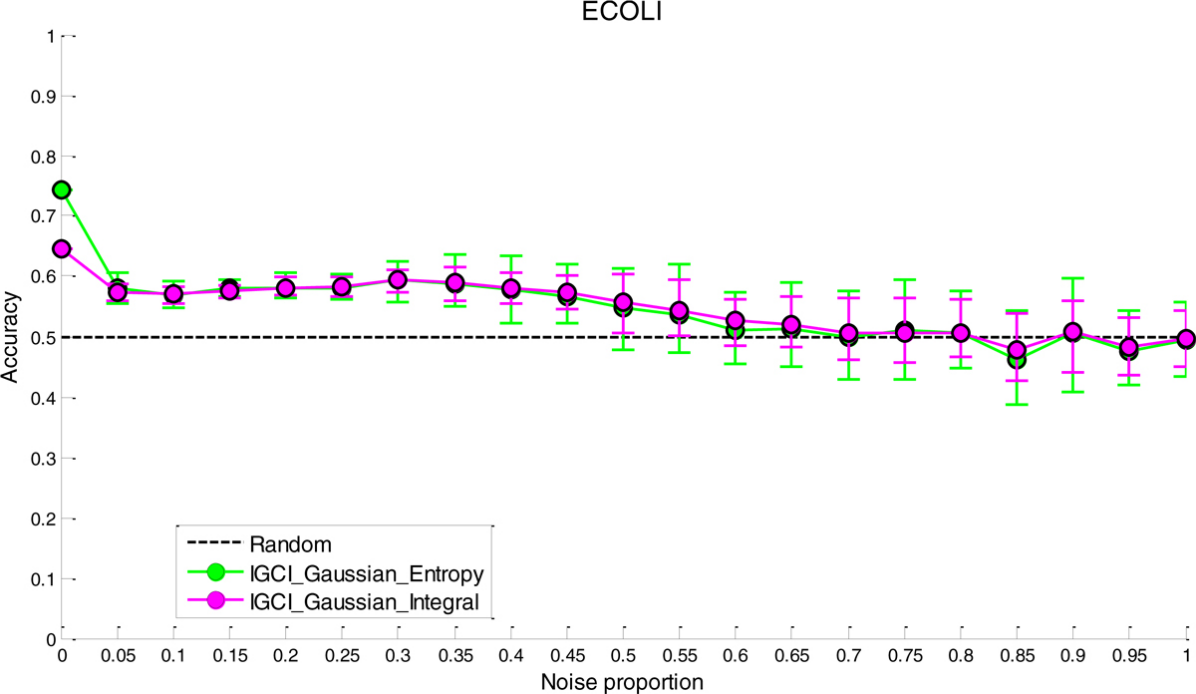
***Sensitivity analysis to noise in ECOLI gold standard for the best two IGCI methods***. Error bars denote 80% intervals of variation that were empirically estimated in 20 datasets for each value of the noise proportion.

**Figure 5:**
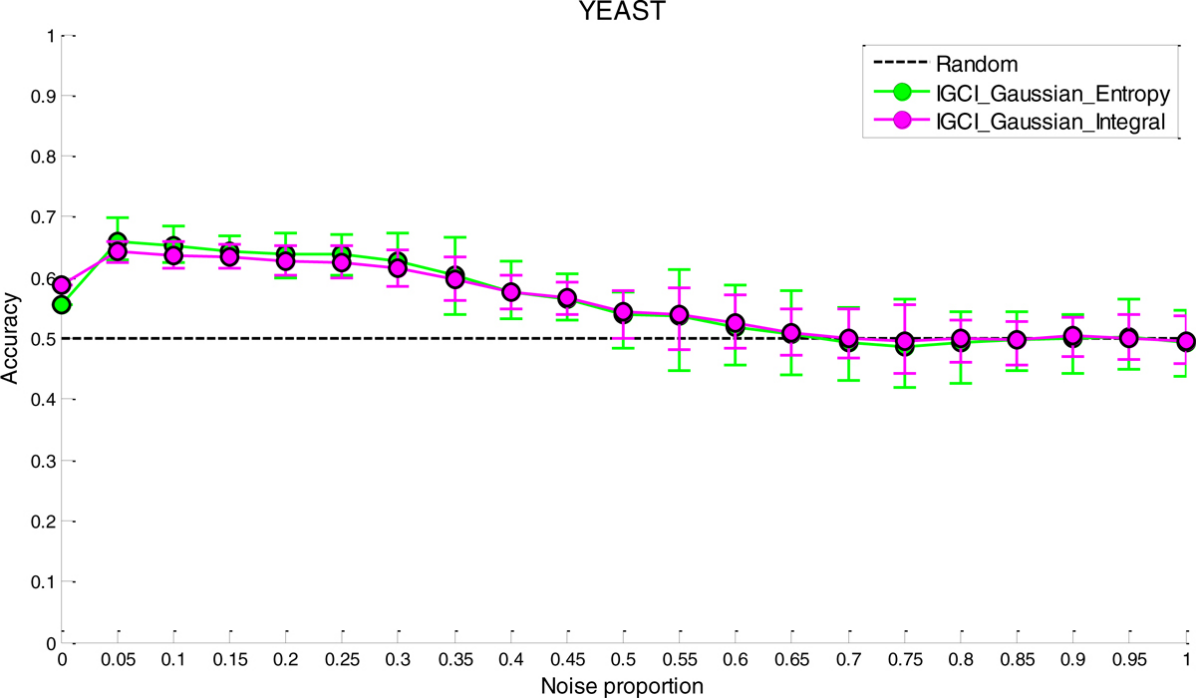
***Sensitivity analysis to noise in YEAST gold standard for the best two IGCI methods***. Error bars denote 80% intervals of variation that were empirically estimated in 20 datasets for each value of the noise proportion.

The results of sensitivity analysis to noise for the two best performing methods (IGCI Gaussian/Entropy and IGCI Gaussian/Integral) are given in Figures 2, 3, 4, 5 for NOTCH1, RELA, ECOLI, and YEAST gold standards, respectively. In all gold standards except for YEAST, the accuracy of the methods decreases with increasing noise proportion. On the other hand, in YEAST gold standard the performance of causal orientation methods significantly *increases *when a small amount of noise is added, and then gradually decreases for higher proportions of noise. The Additional file 1 provides a detailed analysis of this phenomenon.

Whereas in NOTCH1 and RELA gold standards it takes only 5-10% of noise to make the results statistically indistinguishable from orientation by chance, in YEAST and ECOLI gold standards the methods can tolerate much higher proportions of noise and still produce statistically significant results. This can be attributed to a larger number of transcription factors in YEAST and ECOLI gold standards, as well as larger sample sizes in the corresponding datasets which both decrease the variability of the results.

A decrease in performance upon the addition of noise is theoretically expected since IGCI assumes a noise-free model, and the addition of Gaussian noise violates its sufficient assumptions. Also, as can be seen in the figures, the IGCI Gaussian/Integral method has lower variance than the IGCI Gaussian/Entropy method. The above results are consistent with our prior findings and statistical significance testing by the exact test (see Figures S1-S4 in the Additional file 1).

### Sensitivity analysis to sample size

**Figure 6:**
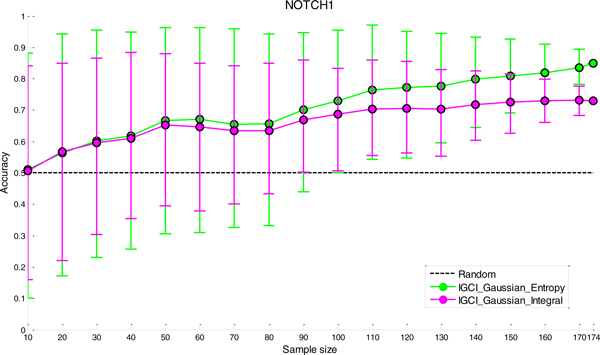
***Sensitivity analysis to sample size in NOTCH1 gold standard for the best two IGCI methods***. Error bars denote 80% intervals of variation that were empirically estimated in 100 sampled datasets of each sample size.

**Figure 7:**
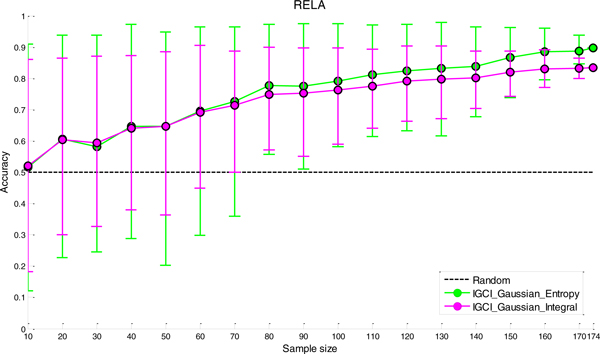
***Sensitivity analysis to sample size in RELA gold standard for the best two IGCI methods***. Error bars denote 80% intervals of variation that were empirically estimated in 100 sampled datasets of each sample size.

**Figure 8:**
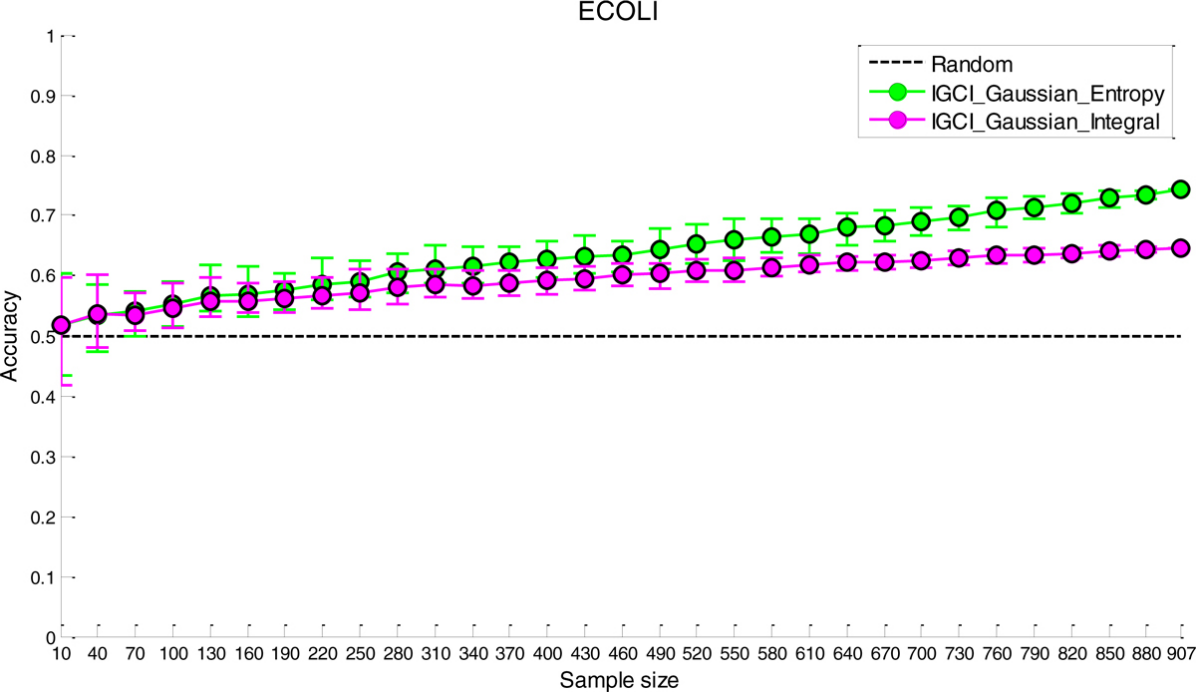
***Sensitivity analysis to sample size in ECOLI gold standard for the best two IGCI methods***. Error bars denote 80% intervals of variation that were empirically estimated in 20 sampled datasets of each sample size.

**Figure 9:**
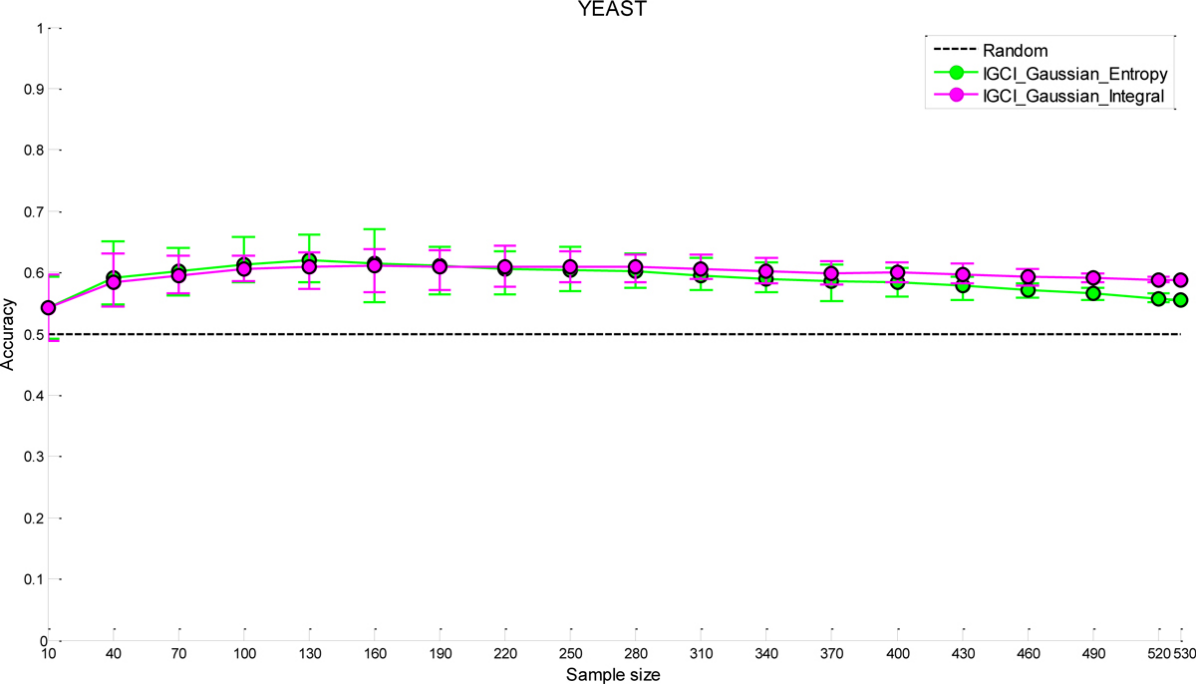
***Sensitivity analysis to sample size in YEAST gold standard for the best two IGCI methods***. Error bars denote 80% intervals of variation that were empirically estimated in 100 sampled datasets of each sample size.

The results of sensitivity analysis to sample size for the two best performing methods (IGCI Gaussian/Entropy and IGCI Gaussian/Integral) are given in Figures 6, 7, 8, 9 for NOTCH1, RELA, ECOLI, and YEAST gold standards, respectively. In all gold standards except for YEAST, the accuracy of the methods decreases as the sample size gets smaller. On the other hand, in YEAST gold standard the performance of causal orientation methods slightly increases by reducing the sample size and then gradually decreases for smaller sample sizes. The Additional file 1 provides a detailed analysis of this phenomenon.

Whereas in NOTCH1 and RELA gold standards results become statistically indistinguishable from orientation by chance when the sample size is <80-100, in YEAST and ECOLI gold standards the methods yield statistically significant results for smaller sample sizes. This can be attributed to a larger number of transcription factors in YEAST and ECOLI gold standards which decreases variability of the results.

Also, as can be seen in the figures, the IGCI Gaussian/Integral method has lower variance than the IGCI Gaussian/Entropy method. The above results are consistent with our prior findings in statistical significance testing by the exact test (see Figures S1-S4 in the Additional file 1).

### Ensemble causal orientation

For each gold standard, Table 9 compares the AUC achieved by the best individual causal orientation method to the AUC achieved by the ensemble method, which combines the predictions of all 11 methods using a logistic regression model. A detailed description of the ensemble modeling methodology is given in the Methods section. As can be seen, ensemble causal orientation achieves higher values of AUC than any individual causal orientation method in all four gold standards. It is worthwhile to highlight the magnitude of the improvement in the YEAST gold standard: the ensemble approach improves performance over the best individual causal orientation method (IGCI Gaussian/Integral) by 0.164 AUC. Table 10 provides coefficients for the ensemble logistic regression model in the YEAST gold standard. Bold values correspond to coefficients that are statistically significant at 0.05 alpha level. The model preserves its performance (0.822 AUC) when it is trained/tested using only the 5 causal orientation methods that have statistically significant coefficients. Therefore the improvement in AUC in the YEAST gold standard can be attributed to effectively combining the IGCI and ANM-MML causal orientation predictions by the logistic regression ensemble model.

**Table 9:** Ensemble causal orientation results and comparison with the best performing individual causal orientation methods

	ECOLI	YEAST	NOTCH1	RELA
Best individual causal orientation method (AUC)	0.828	0.658	0.926	0.970
Ensemble method (AUC)	0.837	0.822	0.984	0.992
Improvement (AUC)	*0.009*	*0.164*	*0.058*	*0.022*
Statistical significance of improvement (p-value)	0.3407	**<0.0001**	**0.0062**	**<0.0001**

Bold p-values indicate a statistically significant performance improvement by using an ensemble causal orientation. The p-values were obtained from Delong's test for AUC comparison [45].

**Table 10:** Coefficients for the ensemble logistic regression model trained in the YEAST gold standard

Method (feature in the logistic regression model)	Beta	P-value
ANM	-1.20	0.291
PNL	-0.27	0.750
IGCI (Uniform/Entropy)	**-128.03**	**<0.0001**
IGCI (Uniform/Integral)	**135.07**	**<0.0001**
IGCI (Gaussian/Entropy)	**99.20**	**<0.0001**
IGCI (Gaussian/Integral)	**-106.45**	**<0.0001**
GPI-MML	1.15	0.578
ANM-MML	**-9.87**	**0.017**
GPI	1.45	0.298
ANM-GAUSS	0.40	0.808
LINGAM	0.11	0.963

Bold values correspond to coefficients that are statistically significant at 0.05 alpha level. We note that due to multicollinearity among the IGCI Uniform methods and among the IGCI Gaussian methods, care must be taken when interpreting the logistic regression coefficients [36].

The above results were obtained by holdout validation where we used different portions of the same gold standard for training and testing ensemble models. We also experimented with training and testing ensemble models on different gold standards. First we experimented with the RELA and NOTCH1 gold standards that were derived from the same organism and phenotype, and thus are likely to be distributionally similar and support cross-gold standard application of the ensemble model. We find that an ensemble logistic regression model trained on RELA obtains AUC = 0.996 when tested on NOTCH1, and likewise an ensemble model trained on NOTCH1 obtains AUC = 0.989 when tested on RELA. Both these results significantly improve performance over the best individual causal orientation method (IGCI Gaussian/Entropy) in both NOTCH1 and RELA gold standards (with p-values <0.0001).

In addition, we experimented with the YEAST and ECOLI gold standards which originate from different organisms and thus are unlikely to be distributionally similar; for this reason they *a priori *question cross-gold standard application of the ensemble model. Indeed, our results confirm this expectation: an ensemble logistic regression model trained on YEAST performs with AUC = 0.4833 in ECOLI, and an ensemble model trained in ECOLI performs with AUC = 0.5916 in YEAST. Neither of these results improves the best individual causal orientation method in the respective gold standard. This suggests that the success of cross-gold standard application of ensemble models is grounded on similarity of the underlying distributions.

## Discussion

This work represents the first comprehensive effort to evaluate performance and furthermore enhance the recently introduced causal orientation methods [13-18] in genomics data. One of the main challenges is the limited availability of gold standards of causal molecular interactions. That is why we have focused on regulatory interactions between transcriptions factors and their target genes that have been recently identified on a genome-wide level in model organisms and human cell lines. These interactions have a well-defined causal directionality (from a transcription factor to its target gene) and can be readily used for an evaluation study such as ours. However, it is possible that some edges in the gold standards (especially, NOTCH1 and RELA) have causal relationships in both directions due to feedback mechanisms. Since the signal in the direction from a transcription factor to its target gene is expected to be stronger than in the opposite direction (due to attenuation in the signal transduction pathways), we are implicitly assuming that in such cases a causal orientation method would prefer the direction from transcription factor to gene. If this assumption is not true, this does not invalidate results of the methods (because the direction from transcription factor to gene is valid) but provides additional explanations as to why some methods prefer the opposite causal direction.

Even though the choice of the gold standard with transcription factor-gene regulatory interactions enables this study, its practical relevance may be limited in the organisms/settings where all transcription factors have already been identified. That is why we plan to work on extending this evaluation to other types of causal molecular interactions, for example in cellular protein signaling networks [39].

In this study we have implicitly assumed that unoriented edges (representing causal interactions between a transcription factor and its target gene without specifying which of the two genes is a transcription factor and which is its regulatory target) are given by an Oracle and we have evaluated performance of only causal orientation methods. However, in practical tasks one typically has to both discover and orient edges. Although we have previously evaluated methods for discovery of unoriented edges [12], it will be interesting to assess the performance of the two classes of methods (for edge discovery and for its orientation) when they are applied together.

Finally, we think that a fruitful area of research will be to extend this study by comparison with classical causal orientation techniques that output Markov equivalence classes of graphs (based on v-structures with constraint propagation) and thus, in general, can orient only a subset of edges in the graph [1].

## Conclusions

In this paper we have taken a first step toward practical use of recent causal orientation techniques in the genomics domain. First of all, we report results of an extensive study of causal orientation methods in genomics data that utilized 12 methods/variants to distinguish cause (transcription factor) from effect (target gene) in 5,739 causal interactions. We have found that IGCI Gaussian methods [16,17] often accurately infer directionality of the causal interaction, and they outperform other causal orientation techniques. In addition, we have performed sensitivity analyses that allow us to empirically establish the minimal requirements for the sample size and maximal level of noise that can be tolerated by the best performing causal orientation techniques. Second, we described a novel ensemble technique for causal orientation that combines decisions of individual causal orientation methods to provide a more powerful predictor of causal directionality. The proposed ensemble method was found to be more accurate than any best individual causal orientation method in the tested data. In summary, our results suggest that causal orientation methods have significant potential to facilitate reconstruction of molecular pathways by minimizing the number of required randomized experiments to find causal directionality and by avoiding experiments that are infeasible and/or unethical.

## Competing interests

The authors declare that they have no competing interests.

## Authors' contributions

AS conceived the study. All authors participated in the design of the study. MH and NIL performed all computational experiments. AS and MH drafted the manuscript. All authors edited, read, and approved the final manuscript.

## Supplementary Material

Additional file 1 **This file contains (1) brief description of causal orientation algorithms; (2) results of causal orientation methods ANM, PNL, and GPI obtained by assessing statistical significance of the forward and backward causal models; (3) detailed results of significance testing of IGCI Gaussian/Entropy and Gaussian/Integral methods; (4) explanation of performance increase due to adding small amount of noise or reducing the sample size in YEAST gold standard**. Click here for file
