# Supplementary material for: New methods for separating causes from effects in genomics data
Source: BMC Genomics. 2012 Dec 17;13(Suppl 8):S22. doi: 10.1186/1471-2164-13-S8-S22 (PMC3535696; doi:10.1186/1471-2164-13-S8-S22)
Supplement: Additional file 1 — This file contains (1) brief description of causal orientation algorithms; (2) results of causal orientation methods ANM, PNL, and GPI obtained by assessing statistical significance of the forward and backward causal models; (3) detailed results of significance testing of IGCI Gaussian/Entropy and Gaussian/Integral methods; (4) explanation of performance increase due to adding small amount of noise or reducing the sample size in YEAST gold standard. [file 1471-2164-13-S8-S22-S1.pdf]

Appendix

Brief description of causal orientation algorithms

- ***ANM***: Perform non-linear regression of Y on X to get an estimate f^* of f. Calculate the residual Y - f*(X), and test if it is independent of X using a kernelized statistical independence test (HSIC) to obtain a p-value p_1. Repeat the same process in the opposite direction to obtain a p-value p_2. If $p_1 > p_2$ conclude X → Y, otherwise X ← Y.
- ***PNL***: First reformulate the data generating process as a non-linear ICA model where one of the sources is the noise term e_1 (assuming the model $Y = f_2(f_1(X) + e_1)$). The noise term is recovered by solving an optimization problem which finds non-linearities that make the outputs as independent as possible by minimizing their mutual information. Test if the noise term e_1 is independent of X using a kernelized statistical independence test (HSIC) to obtain a p-value p_1. Repeat the same process in the opposite direction for Y and noise term e_2 (assuming the model $X = g_2(g_1(Y) + e_2)$) to obtain a p-value p_2. If $p_1 > p_2$ conclude X → Y, otherwise X ← Y.
- ***IGCI***: Estimate the difference in KL-divergences of P(X) and P(Y) with respect to the reference distributions by either estimating the divergences separately and computing their difference, or by using an integral-based approximation which directly estimates their difference. If the resulting difference is negative, conclude X → Y, otherwise X ← Y.
- ***GPI-MML***: Estimate P(X) by assuming a Gaussian mixture model as a prior distribution of X and marginalizing out the parameters. The resulting integral is approximated using a Minimum Message Length technique. Then estimate P(Y|X) by marginalizing over noise and function parameters, and approximating the resulting integral using non-linear optimization techniques. Combining these two quantities gives the likelihood of the observed data given X→Y: DL(X→Y) = -log(P(X)) -

log(P(Y|X)). Repeat the process, this time estimating P(Y) and P(X|Y), to obtain DL(X←Y). If DL(X→Y) < DL(X←Y), conclude X → Y, otherwise X ← Y.

- ***ANM-MML***: Same as GPI-MML, except for a different method of estimating P(Y | X), where the covariance matrix used in the Gaussian process is constant with respect to the noise (which reflects the additive noise assumption). As before, we obtain the likelihood of the observed data given X→Y: DL(X→Y) = -log(P(X)) - log(P(Y|X)). Repeating the process, this time estimating P(Y) and P(X|Y), we obtain DL(X ←Y). If DL(X→Y) < DL(X←Y), conclude X → Y, otherwise X ← Y.
- ***GPI***: Similar to ANM, only we perform non-linear regression of Y on X *and* e. Since e is supposed to represent all the unobserved causes as well as noise, this can be thought of as accounting for latent variables. Estimate the noise e from the equation Y = f*(X,e), and test if it is independent of X using a kernelized statistical independence test (HSIC) to obtain a p-value p_1. Repeat the same process in the opposite direction to obtain a p-value p_2. If $p_1 > p_2$ conclude X → Y, otherwise X ← Y.
- ***ANM-GAUSS***: Same as ANM-MML, except for a different method of estimating P(X) using a single Gaussian, rather than a mixture model. As before, we obtain the likelihood of the observed data given X→Y: DL(X→Y) = -log(P(X)) - log(P(Y|X)). Repeating the process, this time estimating P(Y) and P(X|Y), we obtain DL(X←Y). If DL(X→Y) < DL(X←Y), conclude X → Y, otherwise X ← Y.
- ***LINGAM***: Estimate a model of the form $Y=b_2X+e_1$ and $X=b_1Y+e_2$, where e_1 and e_2 are independent, using independent component analysis (ICA). If $b_1 < b_2$, conclude X → Y, otherwise X ← Y.

Results of causal orientation methods ANM, PNL, and GPI obtained by assessing statistical significance of the forward and backward causal models

Recall that all causal relations in the gold standard are of the type TF → G ("TF" stands for a transcription factor and "G" stands for its target gene). The tables below adopt the following notation:

- **TF → G**: Number of times the method discovers that the model TF → G is statistically significant, while the model TF ← G is not statistically significant (at the given alpha level).
- **TF ← G**: Number of times the method discovers that the model TF ← G is statistically significant, while the model TF → G is not statistically significant (at the given alpha level).
- **TF ↔ G**: Number of times the method discovers that both models TF ← G and TF → G are statistically significant (at the given alpha level).
- **TF G**: Number of times the method discovers that neither model TF ← G and TF → G is statistically significant at the given alpha level.
- **Accuracy***: Accuracy for confident decisions only, that is computed as:
  TF → G / [TF → G + TF ← G].

ECOLI results:

Method	TF → G	TF ← G	TF ↔ G	TF G	Accuracy*
ANM (α = 0.01)	53	60	5	1489	0.469
ANM (α = 0.05)	24	25	2	1556	0.490
ANM (α = 0.10)	13	15	0	1579	0.464
PNL (α = 0.01)	172	226	135	1074	0.432
PNL (α = 0.05)	108	160	65	1274	0.403
PNL (α = 0.10)	87	138	30	1352	0.387
GPI (α = 0.01)	120	82	12	1393	0.594
GPI (α = 0.05)	55	40	2	1510	0.579
GPI (α = 0.10)	28	15	1	1563	0.651

YEAST results:

Method	TF → G	TF ← G	TF ↔ G	TF G	Accuracy*
ANM (α = 0.01)	284	507	230	1627	0.359
ANM (α = 0.05)	200	350	104	1994	0.364
ANM (α = 0.10)	148	265	68	2167	0.358
PNL (α = 0.01)	432	518	569	1129	0.455
PNL (α = 0.05)	354	425	280	1589	0.454
PNL (α = 0.10)	281	337	154	1876	0.455
GPI (α = 0.01)	322	557	356	1413	0.366
GPI (α = 0.05)	222	385	153	1888	0.366
GPI (α = 0.10)	170	274	90	2114	0.383

NOTCH1 results:

Method	TF → G	TF ← G	TF ↔ G	TF G	Accuracy*
ANM (α = 0.01)	29	71	449	4	0.290
ANM (α = 0.05)	79	117	341	16	0.403
ANM (α = 0.10)	98	156	260	39	0.386
PNL (α = 0.01)	32	18	499	4	0.640
PNL (α = 0.05)	69	39	433	12	0.639
PNL (α = 0.10)	90	69	369	25	0.566
GPI (α = 0.01)	14	32	499	8	0.304
GPI (α = 0.05)	54	55	411	33	0.495
GPI (α = 0.10)	81	75	339	58	0.519

RELA results:

Method	TF → G	TF ← G	TF ↔ G	TF G	Accuracy*
ANM (α = 0.01)	13	112	802	4	0.104
ANM (α = 0.05)	41	170	696	24	0.194
ANM (α = 0.10)	60	212	611	48	0.221
PNL (α = 0.01)	37	22	868	4	0.627
PNL (α = 0.05)	83	68	766	14	0.550
PNL (α = 0.10)	120	105	673	33	0.533
GPI (α = 0.01)	25	38	856	12	0.397
GPI (α = 0.05)	66	83	729	53	0.443
GPI (α = 0.10)	84	110	642	95	0.433

Detailed results of significance testing of IGCI Gaussian/Entropy and Gaussian/Integral methods

Figures S1-S4 below report causal orientation performance (measured by the AUC or accuracy metric) of IGCI methods (Gaussian/Entropy or Gaussian/Integral) in real data (red line in the graphs and second column in the tables) and in 1,000 random datasets from Normal distribution with mean 0 and standard deviation 1 (blue histograms), as well as empirical probability of observing higher performance in the random data than the observed performance in the real data (third column in the tables).

- Figure S1: IGCI Gaussian/Entropy method assessed with the accuracy metric.
- Figure S2: IGCI Gaussian/Entropy method assessed with the AUC metric.
- Figure S3: IGCI Gaussian/Integral method assessed with the accuracy metric.
- Figure S4: IGCI Gaussian/Integral method assessed with the AUC metric.

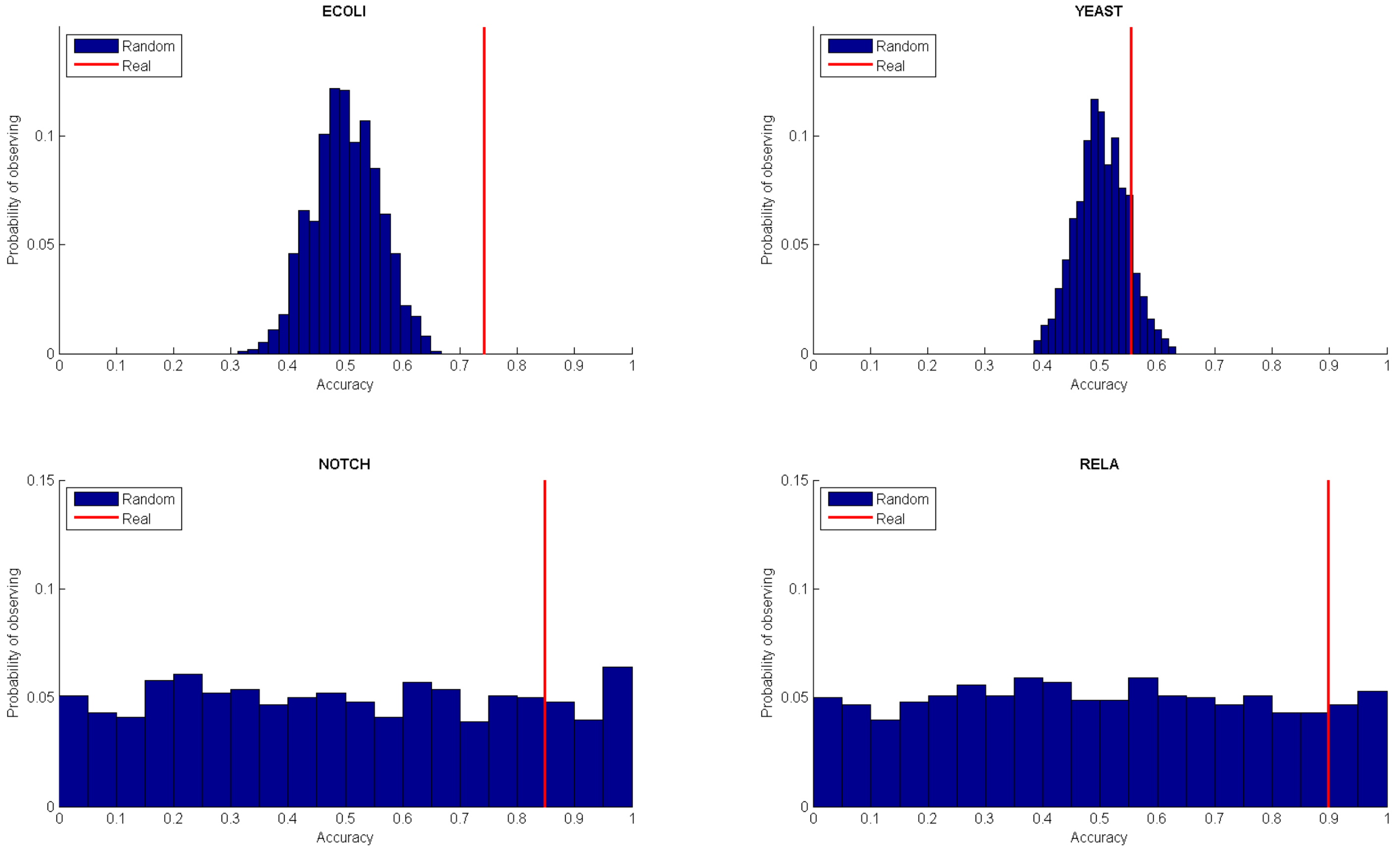

Dataset	Accuracy	P-value
ECOLI	0.742	0
YEAST	0.555	0.115
NOTCH	0.848	0.155
RELA	0.898	0.101

Figure S1

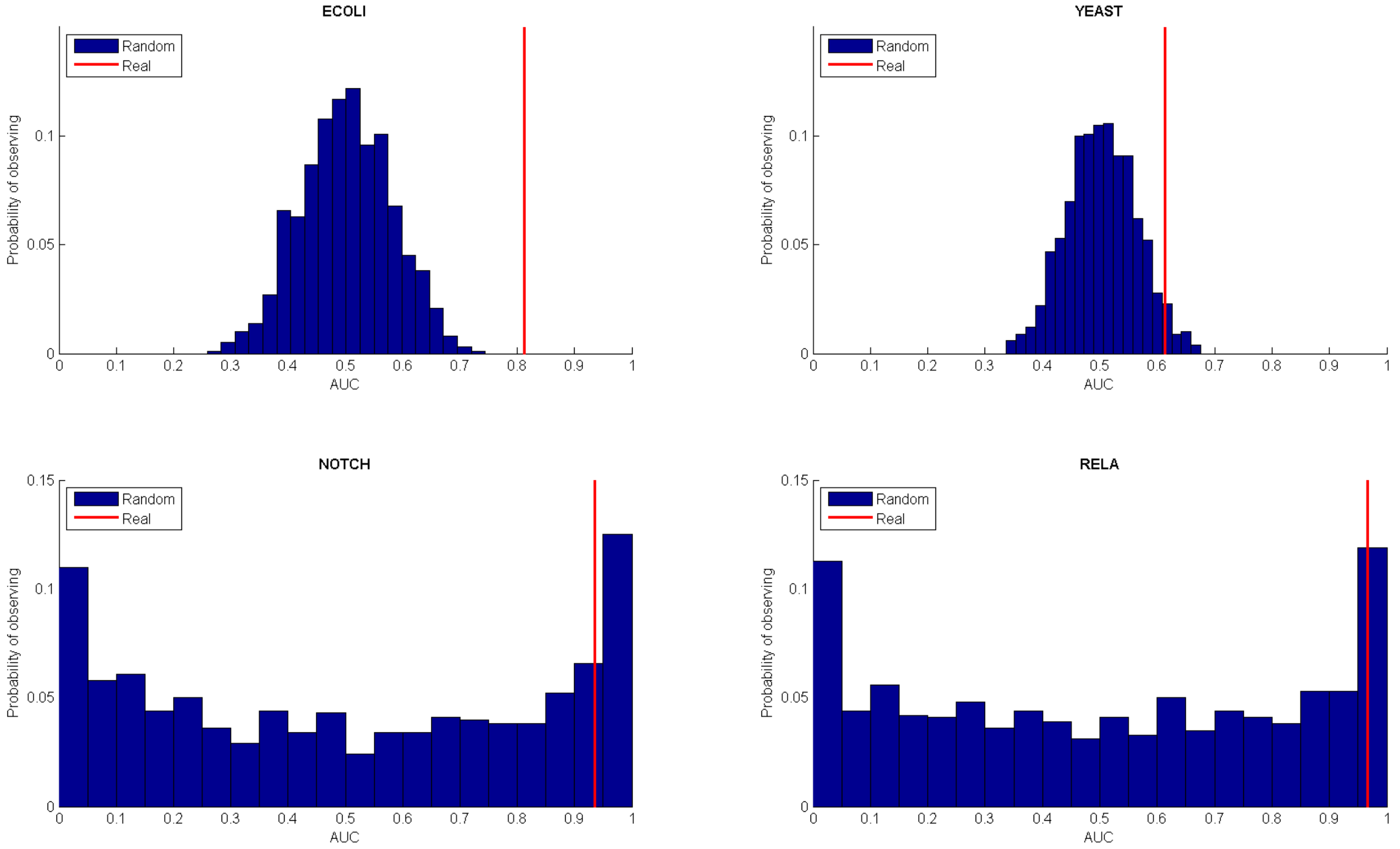

Dataset	AUC	P-value
ECOLI	0.813	0
YEAST	0.613	0.037
NOTCH	0.935	0.147
RELA	0.967	0.103

Figure S2

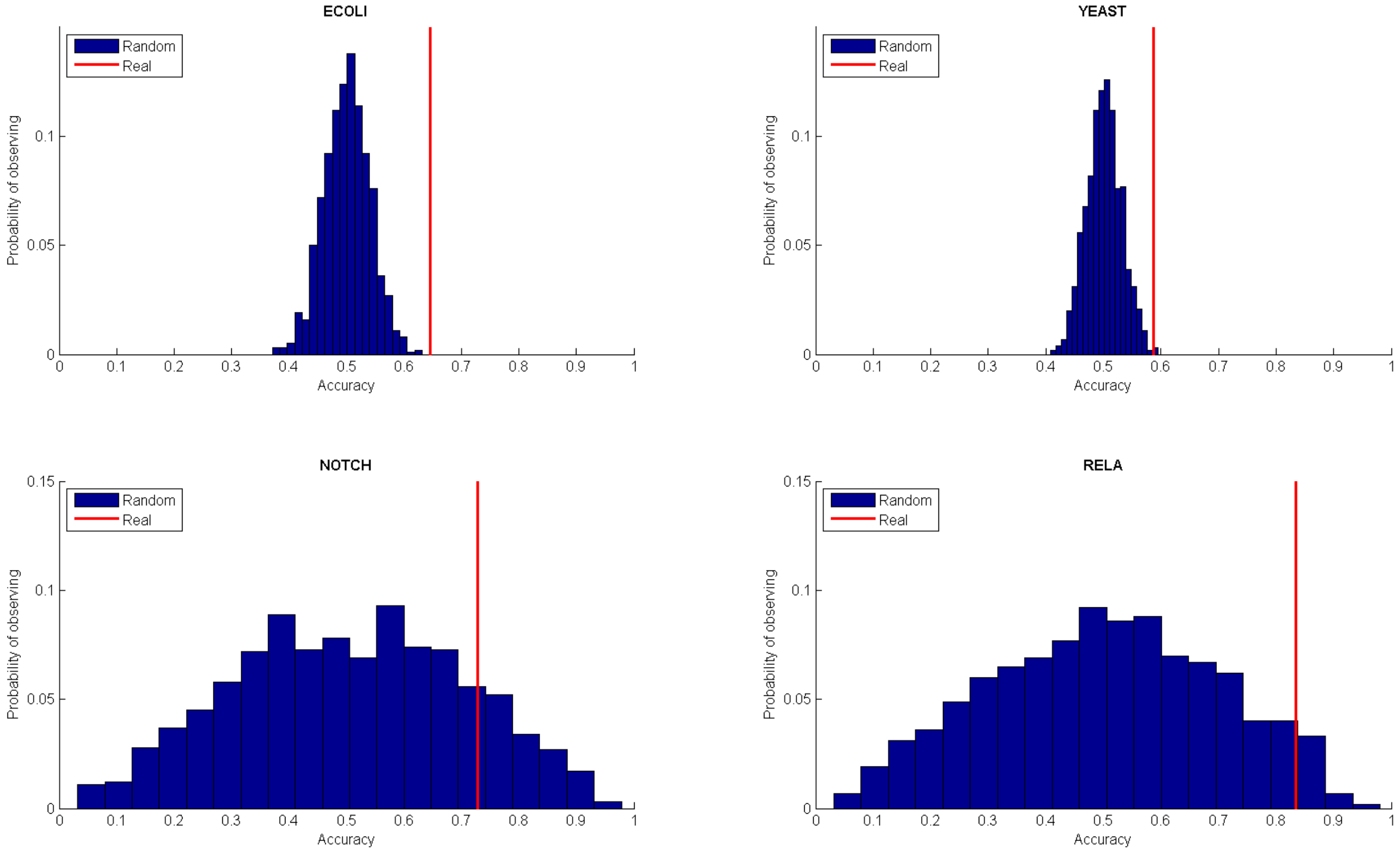

Dataset	Accuracy	P-value
ECOLI	0.645	0
YEAST	0.587	0.003
NOTCH	0.729	0.152
RELA	0.835	0.045

Figure S3

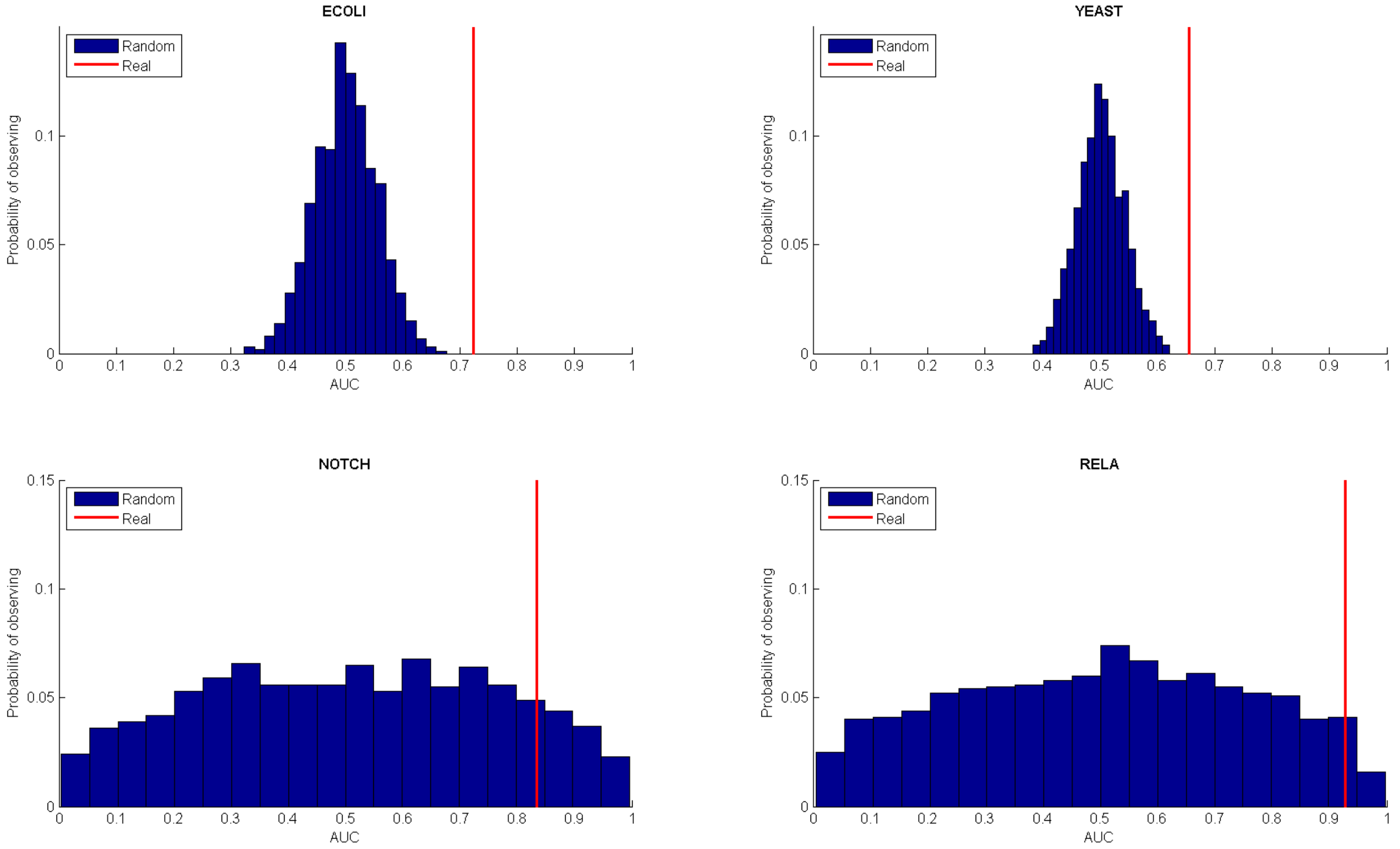

Dataset	AUC	P-value
ECOLI	0.724	0
YEAST	0.655	0
NOTCH	0.834	0.122
RELA	0.927	0.031

Figure S4

Performance increase due to adding small amount of noise or reducing the sample size in YEAST gold standard

Performance increase due to adding noise: We have plotted output scores of the IGCI methods for each transcription factor both with and without added noise. The plots for 5%, 10%, 20%, 30%, 40%, and 50% of noise are given in Figures S5-S10. To interpret these figures we remind the readers that the negative scores correspond to correct orientations, whereas positive scores correspond to incorrect orientations. As can be seen, adding noise causes both negative and positive scores (corresponding to correct and incorrect predictions, respectively) to converge to zero, as expected. However, as we increase the noise level, the IGCI outputs for the cause-effect pairs that have been correctly predicted in the noiseless data (i.e., have negative scores) converge to zero slower than the IGCI outputs for the cause-effect pairs that have been incorrectly predicted in the noiseless data (i.e., have positive scores). As a result, for small amounts of noise, most correct predictions in the noiseless data are retained (they still have negative scores) while the incorrect predictions increasingly behave like random. Overall, this results in an increase of accuracy.

For example, assume that we have 100 cause-effect pairs and IGCI correctly predicted 80 of them in the noiseless data, resulting in 80% accuracy. Then with the addition of a small amount of noise, we retain 80 correct predictions while the 20 other predictions are now classified randomly, resulting in 10 correct and 10 incorrect. Overall, this leads to 90% accuracy, so we have a 10% increase.

- Figure S5: Scores for each cause-effect pair in YEAST gold standard obtained using the IGCI Gaussian/Entropy method in the data with 5% noise. Cyan points correspond to the IGCI output scores in the noiseless data. Grey points correspond to the IGCI output scores for each of the 20 noisy datasets. Magenta points correspond to the average IGCI output scores over all noisy

datasets. The results are plotted based on sorting the IGCI output scores in the noiseless data; that is why the cyan points are monotonically increasing.

- Figure S6: Scores for each cause-effect pair in YEAST gold standard obtained using the IGCI Gaussian/Entropy method in the data with 10% noise.
- Figure S7: Scores for each cause-effect pair in YEAST gold standard obtained using the IGCI Gaussian/Entropy method in the data with 20% noise.
- Figure S8: Scores for each cause-effect pair in YEAST gold standard obtained using the IGCI Gaussian/Entropy method in the data with 30% noise.
- Figure S9: Scores for each cause-effect pair in YEAST gold standard obtained using the IGCI Gaussian/Entropy method in the data with 40% noise.
- Figure S10: Scores for each cause-effect pair in YEAST gold standard obtained using the IGCI Gaussian/Entropy method in the data with 50% noise.

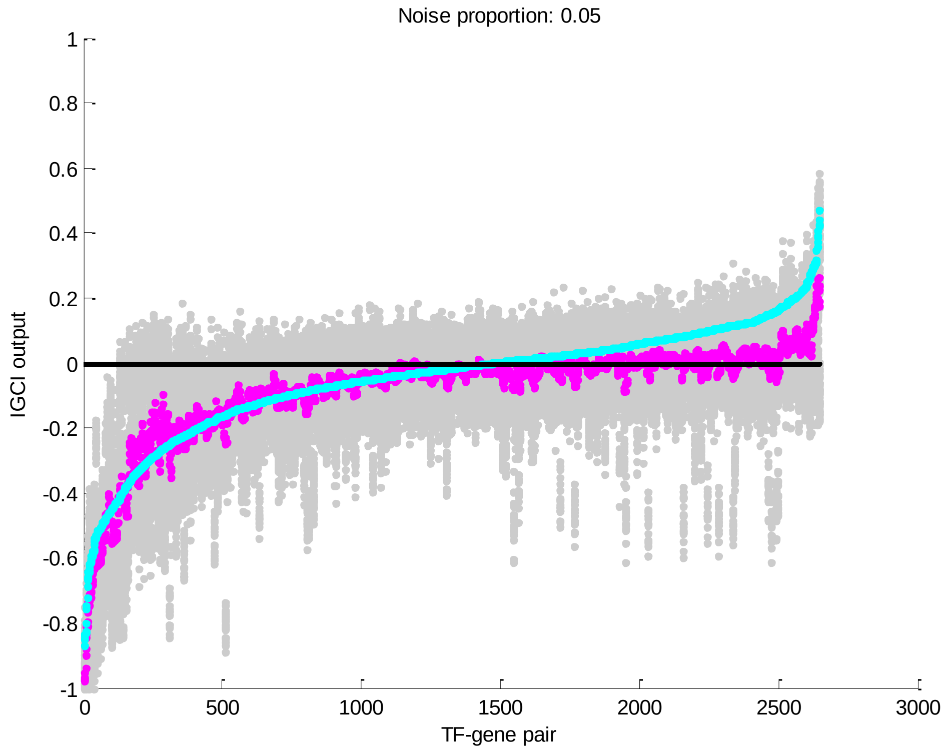

Figure S5

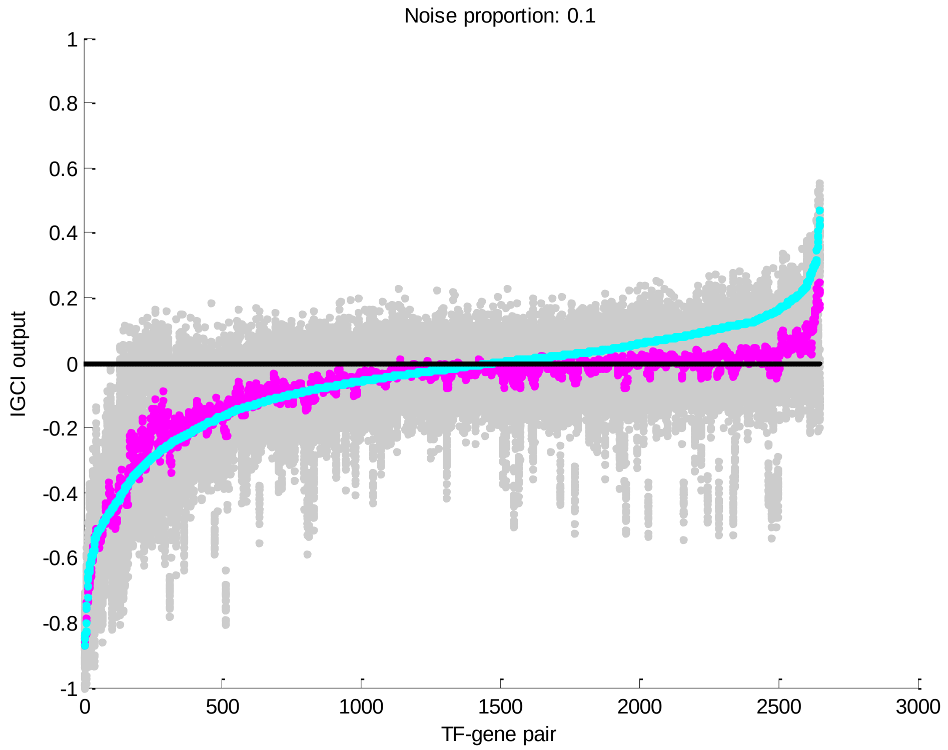

Figure S6

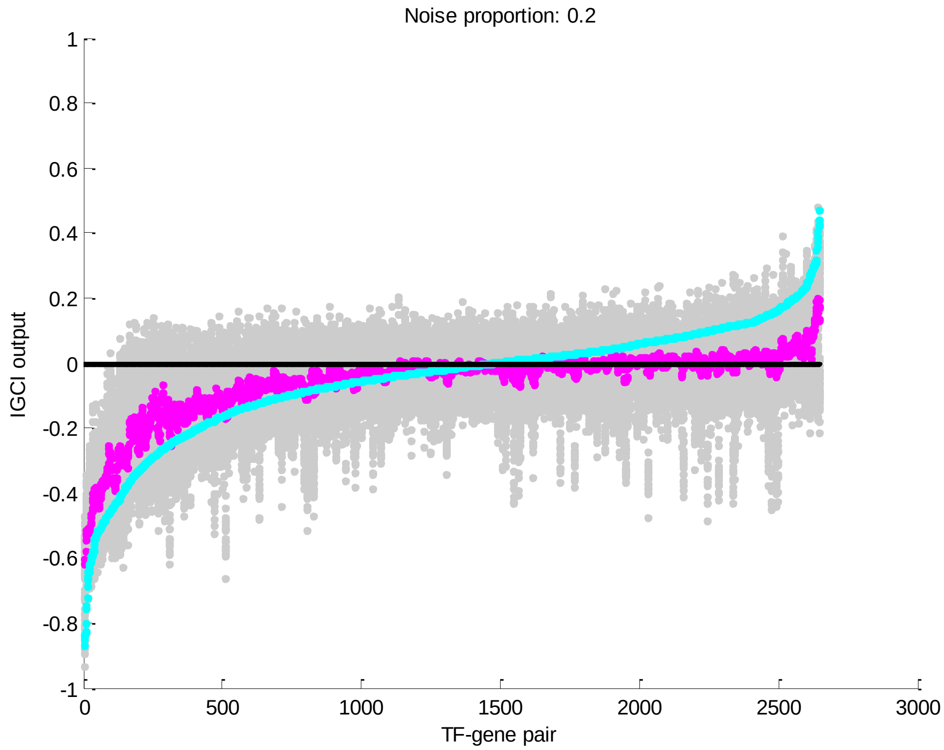

Figure S7

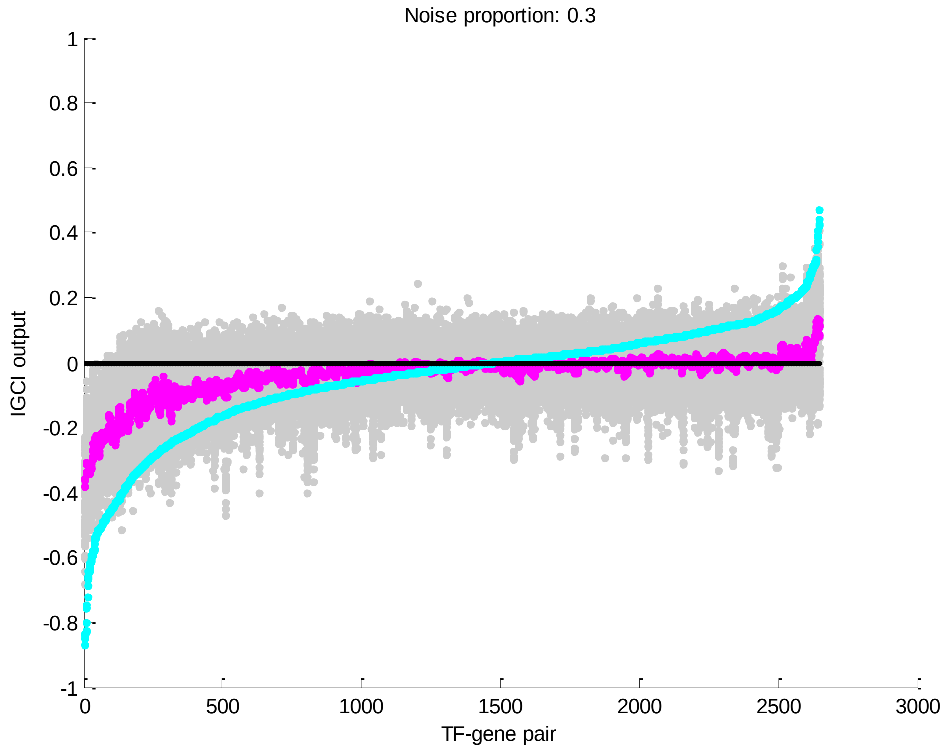

Figure S8

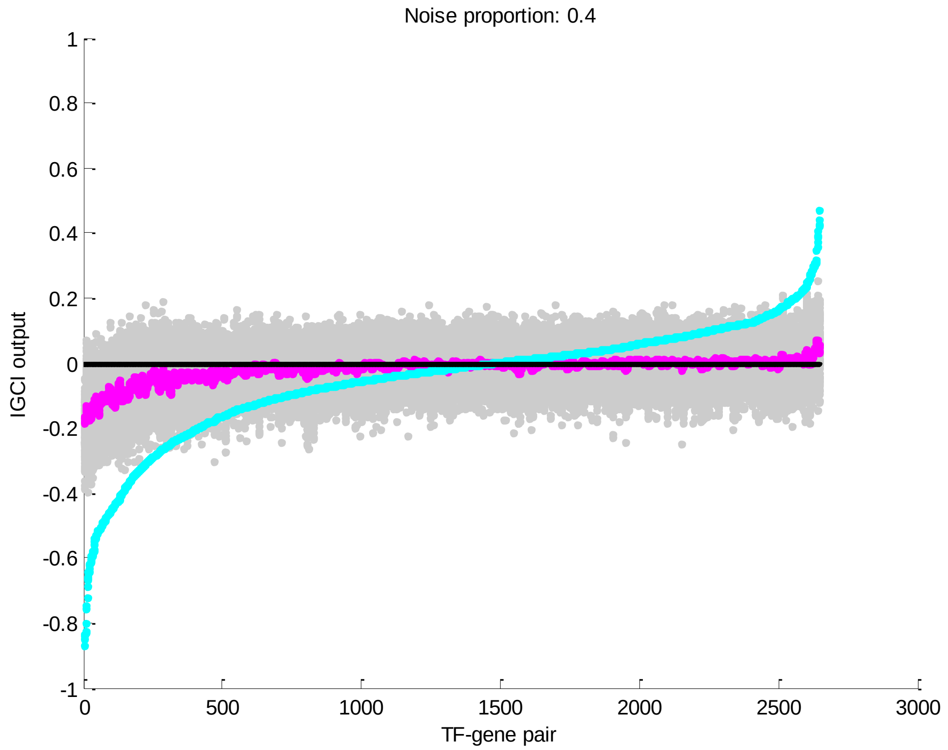

Figure S9

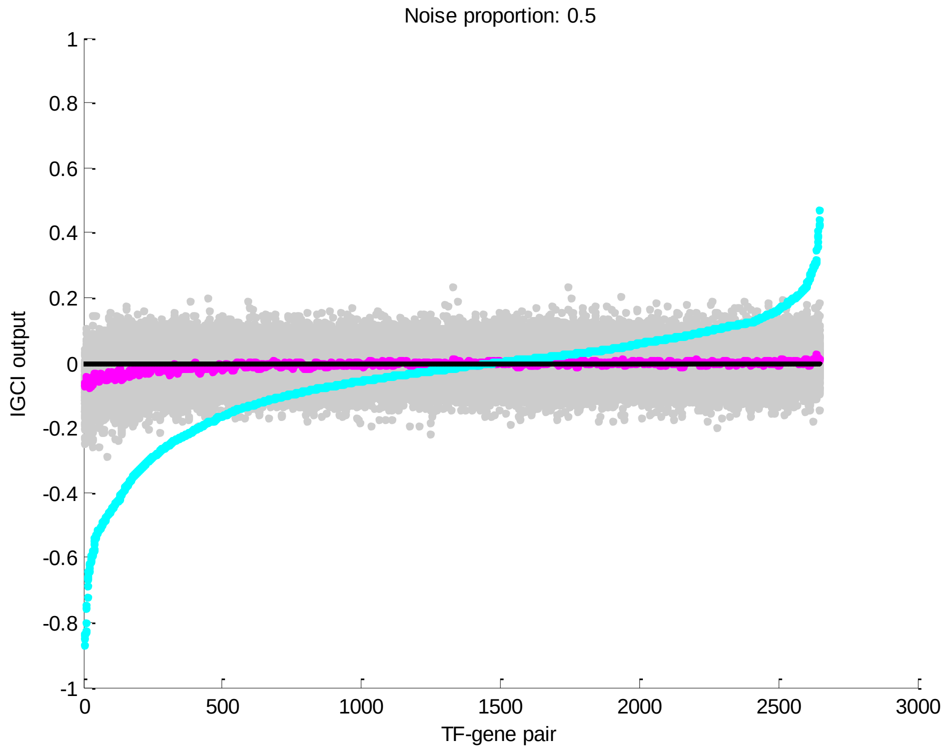

Figure S10

Performance increase due to reducing sample size: We plotted similar graphs to those described above for sample sizes 310, 220, 130 and 40. Decreasing the sample size causes both negative and positive scores (corresponding to correct and incorrect predictions, respectively) to converge to zero, as expected. However, as we decrease the sample size, the IGCI outputs for the cause-effect pairs that have been correctly predicted using all samples (i.e., have negative scores) converge to zero slower than the IGCI outputs for the cause-effect pairs that have been incorrectly predicted using all samples (i.e., have positive scores). As a result, for certain sample sizes, most correct predictions in the full sample data are retained (they still have negative scores), while the incorrect predictions increasingly behave like random. Overall, this results in an increase of accuracy.

- Figure S11: Scores for each cause-effect pair in YEAST gold standard obtained using the IGCI Gaussian/Entropy method in the data using 310 samples. Cyan points correspond to the IGCI output scores using all 530 samples. Grey points correspond to the IGCI output scores for each of the 20 datasets of size 310. Magenta points correspond to the average IGCI output scores over all 20 sampled datasets of size 310. The results are plotted based on sorting of the IGCI output scores in the full sample data; that is why cyan points are monotonically increasing.
- Figure S12: Scores for each cause-effect pair in YEAST gold standard obtained using the IGCI Gaussian/Entropy method in the data with 220 samples.
- Figure S13: Scores for each cause-effect pair in YEAST gold standard obtained using the IGCI Gaussian/Entropy method in the data with 130 samples.
- Figure S14: Scores for each cause-effect pair in YEAST gold standard obtained using the IGCI Gaussian/Entropy method in the data with 40 samples.

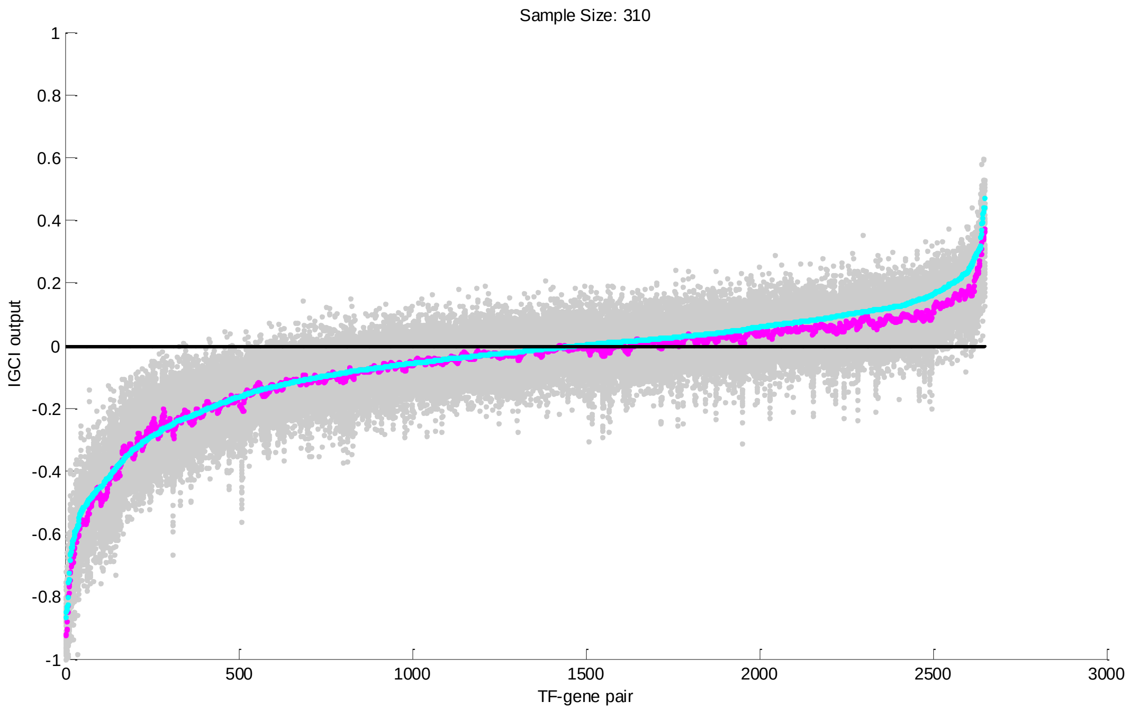

Figure S11

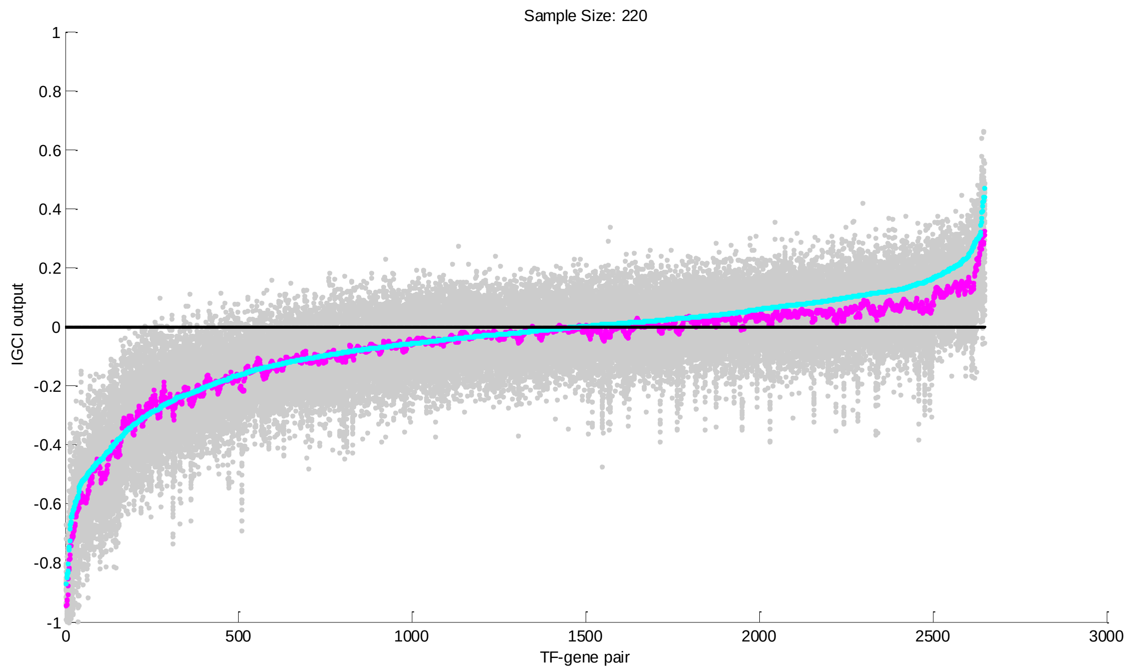

Figure S12

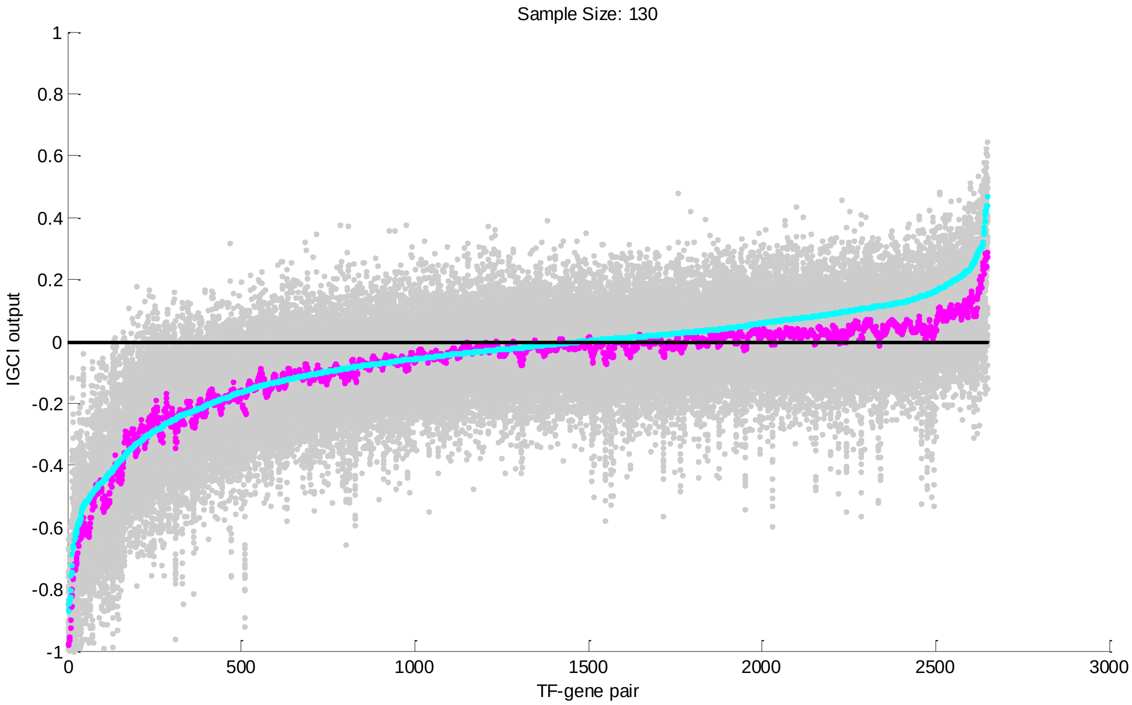

Figure S13

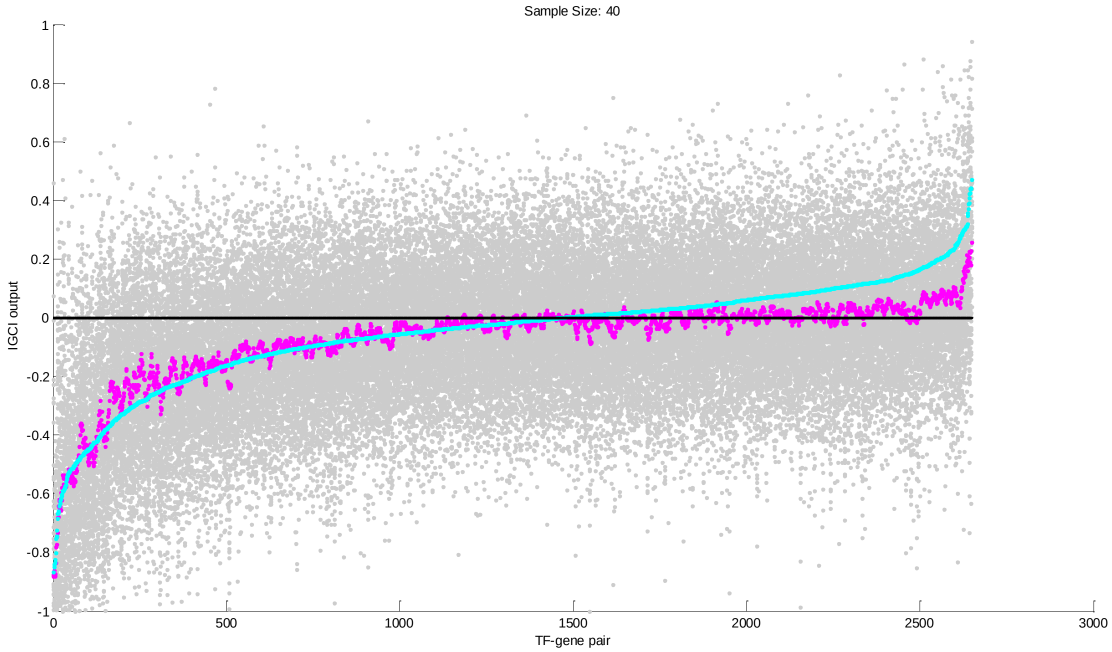

Figure S14
